# Efficacy and safety of TNF inhibitors in the treatment of juvenile idiopathic arthritis: a systematic literature review

**DOI:** 10.1186/s12969-023-00798-8

**Published:** 2023-02-24

**Authors:** Gerd Horneff, Kirsten Minden, Catherine Rolland, Ana C. Hernandez Daly, Cecilia Borlenghi, Nicolino Ruperto

**Affiliations:** 1 Department of General Paediatrics, Asklepios Clinic Sankt Augustin, Sankt Augustin, Germany; 2 Department of Paediatric and Adolescents Medicine, University Hospital of Cologne, Cologne, Germany; 3 German Rheumatism Research Centre Berlin (DRFZ), Leibniz Association, Berlin, Germany; 4 Department of Pediatric Pulmonology, Immunology, and Intensive Care Medicine, Charité – Universitätsmedizin Berlin, Berlin, Germany; 5 Curo Consulting, Envision Pharma Group, Glasgow, UK; 6 Pfizer, Athens, Greece; 7 Pfizer, Buenos Aires, Argentina; 8 IRCCS Istituto Giannina Gaslini, UOSID Centro Trial, Genoa, Italy

## Abstract

**Objective:**

A systematic literature review was conducted to summarize efficacy and safety data from studies that evaluated tumor necrosis factor inhibitors in patients with juvenile idiopathic arthritis (JIA).

**Methods:**

Relevant publications were identified via online searches (cutoff: March 16, 2021). After screening search results, outcome data were extracted if the treatment arm included ≥ 30 patients. Outcomes were described narratively, with efficacy assessed by JIA-American College of Rheumatology (ACR) response criteria and safety assessed by the incidence of serious adverse events (SAEs) per 100 patient-years (100PY).

**Results:**

Among 87 relevant publications included in the qualitative synthesis, 19 publications described 13 clinical trials. Across the 13 trials, the percentages of patients who achieved JIA-ACR30/50/70/90 responses at Week 12 with adalimumab ranged 71–94%, 68–90%, 55–61%, and 39–42%, respectively; with etanercept (Week 12), 73–94%, 53–78%, 36–59%, and 28%; with golimumab (Week 16), 89%, 79%, 66%, and 36%; and with infliximab (Week 14), 64%, 50%, and 22% (JIA-ACR90 not reported). SAE incidence across all time points ranged 0–13.7 SAE/100PY for adalimumab, 0–20.0 SAE/100PY for etanercept, and 10.4–24.3 SAE/100PY for golimumab (1 study). SAE incidence could not be estimated from the 2 infliximab publications.

**Conclusion:**

Tumor necrosis factor inhibitors are effective and well tolerated in the treatment of JIA, but additional evidence from head-to-head studies and over longer periods of time, especially in the context of the transition from pediatric to adult care, would be useful.

**Supplementary Information:**

The online version contains supplementary material available at 10.1186/s12969-023-00798-8.

## Introduction

Juvenile idiopathic arthritis (JIA), the most common rheumatic disease observed in children, has been defined as “arthritis of unknown etiology that begins before the 16th birthday and persists for at least 6 weeks” by the International League of Associations for Rheumatology [1]. Overall, JIA has a reported incidence of 8 per 100,000 and 10–12 per 100,000 in Europe and the USA, respectively, while the corresponding prevalence is 70 per 100,000 and 45–58 per 100,000 [2–4]. In childhood, JIA is a leading cause of short- and long-term disability due to progressive destruction of cartilage and bones within joints, as well as growth retardation [5–8]. Approximately 50% of children who develop JIA continue to have active disease into adulthood, with ongoing physical disability and declining health-related quality of life (QoL) [8–10], but adult patients with JIA may be underrepresented in clinical studies.

As recommended by the treatment guidelines published by the American College of Rheumatology (ACR) [11], first-line pharmacotherapy for JIA usually consists of a combination of nonsteroidal anti-inflammatory drugs (NSAIDs), intra-articular glucocorticoids, and conventional synthetic disease-modifying antirheumatic drugs (csDMARDs), with methotrexate being the most frequently used csDMARD. If a clinically inadequate outcome is achieved with this initial approach, treatment with a biologic DMARD (bDMARD), such as a tumor necrosis factor inhibitor (TNFi), may be considered as a second-line option. However, an International Task Force has proposed replacing this traditional sequential approach to JIA treatment with a treat-to-target strategy, in recognition that remission and low disease activity are currently achievable goals in most patients with JIA with recent therapeutic advances [12].

Two classes of recombinant TNFi are currently used in the treatment of JIA: TNF receptor fusion proteins (e.g., etanercept) and monoclonal anti-TNF antibodies (adalimumab, certolizumab pegol, golimumab, and infliximab), and some related biosimilars [13–21]. Etanercept was the first bDMARD to be evaluated in JIA, followed by infliximab, adalimumab, tocilizumab, abatacept, golimumab, and secukinumab; certolizumab pegol is currently under investigation. To date, no head-to-head trials have compared outcomes with different bDMARDs, especially TNFis, in JIA. Several meta-analyses and systematic reviews of the efficacy and/or safety of these agents have been published [21–27]. We conducted the current systematic literature review (SLR) to provide an update on previous reviews, including more recent literature, and to examine findings from pediatric and adult patients with JIA treated with a TNFi in the context of both clinical trials and large observational studies and registries, which afford experience with treatment in routine care settings. In the following article, we summarize the published data on the efficacy/effectiveness and safety of TNFis when used in clinical trials and observational studies of patients with JIA.

## Methods

### Systematic literature review

The SLR was conducted according to the Preferred Reporting Items for Systematic Reviews and Meta-Analyses (PRISMA) 2009 guidelines (Supplementary Table 1) [28]. The SLR protocol was registered in the PROSPERO database [29].

Relevant articles in English (with no limit on the date of publication) were identified via online searches of the Embase®, MEDLINE®, and PubMed® databases conducted on March 16, 2020, and subsequently updated on March 16, 2021. Relevant abstracts were also identified via online searches of congress websites and abstract supplements covering the most recent meetings (at the time of the search) of the ACR, Childhood Arthritis and Rheumatology Research Alliance, European League Against Rheumatism, and Paediatric Rheumatology European Society.

The searches applied population, intervention, comparator, outcomes, and study types criteria to identify relevant publications (Supplementary Table 2). The search strategies used for each of the online databases are shown in Supplementary Tables 3–5. In addition to online searches, the reference lists of relevant review articles and all publications included in the SLR were also searched by an analyst (C. Rolland) to identify any publications not indexed by the online databases.

### Screening of identified publications

Following the removal of duplicates from the literature search results, 2 analysts (C. Rolland and S. Lucas) independently screened the titles and abstracts of all the identified publications to assess their eligibility according to the inclusion and exclusion criteria (Supplementary Table 6). Any discrepancies between the findings of the analysts were resolved by consultation with a third analyst (V. Young) to provide a majority decision. The same approach involving 2 initial analysts and the resolution of discrepancies via a third analyst was used during all subsequent phases of the SLR (the analysts named above fulfilled the same roles in each phase). Full-text versions of the publications considered eligible for inclusion were obtained and subjected to a second round of screening by applying the same inclusion and exclusion criteria.

### Appraisal of data-source quality

Randomized controlled trials (RCTs) were assessed for bias using the Cochrane Risk of Bias 2 (RoB2) tool [30]. The quality of non-RCTs (nRCTs) and observational studies was assessed using the Newcastle–Ottawa Scale [31]. The appraisals assessed the quality of the clinical trials and not the quality of the publications describing the trials. Congress abstracts were not assessed for quality on the understanding that these publications should be interpreted cautiously because they contain limited information and were subjected to a less stringent peer-review process.

### Data extraction and synthesis

The same 2 analysts who conducted the screening independently extracted relevant data from the included publications using a standardized grid. Data reported from treatment arms that included a total sample of less than 30 patients were not extracted. Data from RCTs, nRCTs, and observational studies were assessed and reported separately in order to account for differences in study design. (Most references for observational studies are available in Additional file 1.)

Key outcomes data were described narratively, with efficacy assessed by JIA-ACR response criteria [32] and Juvenile Arthritis Disease Activity Score [33], when possible, and safety outcomes assessed by the absolute number and incidence of serious adverse events (SAEs). In RCTs, SAEs were defined according to U.S. Food and Drug Administration guidelines as (A) death, (B) life-threatening event, (C) hospitalization or prolongation of hospitalization, (D) persistent or significant disability/incapacity, (E) congenital anomaly or birth defect, and (F) important medical event requiring medical or surgical intervention to prevent serious outcome. The definition of SAEs used in individual observational studies may have varied. The incidence of SAEs was presented as events per 100 patient-years (100PY), when possible. Data from publications reporting SAE rates as events per PY were converted to 100PY using the following calculation: (number of events/total PY exposure) × 100.

## Results

### Literature searches/screening

**Fig. 1:**
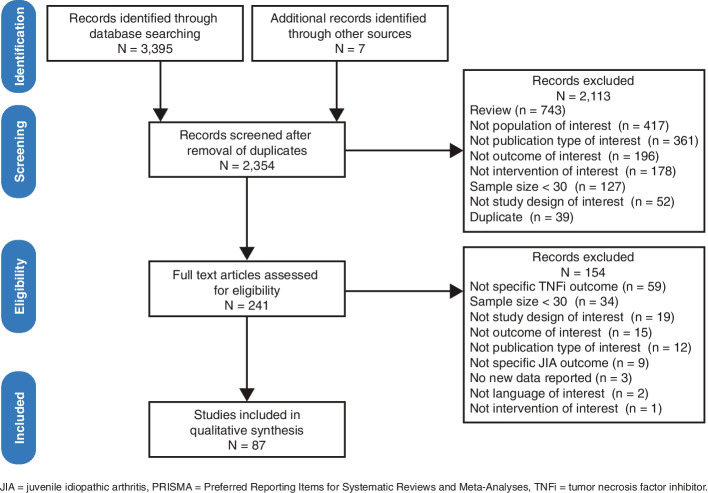
PRISMA diagram of search results

A total of 2,354 relevant publications were identified via the literature searches and screened for eligibility (Fig. 1). After eligibility screening and the removal of duplicates, 87 publications (3.7%) were included in the qualitative synthesis (Table 1; Supplementary Table 7). Most publications were from Europe (*n* = 48); others were multiregional (*n* = 17) or from North America (*n* = 11), Asia (*n* = 3), or South America (*n* = 2).

**Table 1:** Summary of study type and patient characteristics in SLR-identified publications (*n* = 87) by TNFi treatment

	**Adalimumab**	**Etanercept**	**Golimumab**	**Infliximab**
Study type, *n*				
Total	26	67	7	14
Observational	22	55	5	12
RCT	3	8	1	1
nRCT	1	4	1	1
JIA category, *n*				
Multiple	9	26	3	5
PA	3	11	2	1
sJIA	1	5	0	0
nsJIA	1	3	0	0
ERA	1	7	0	0
Age at treatment start, years				
Mean	3.0–17.5	3.1–16.2	11.1–13.6	10.6–21.8
Median	4.9–13.9	10.0–21.0	13.0*	11.9*
Follow-up duration				
Mean, years	3.58–3.96	0.5–6.8	1.0*	0
Median, months	51.0–53.7	12.0–35.6	0	51.0–53.7
Range, months	3–120	3–216	7–40	12–120
Person-years	14–1,855	3–6,726	3.5–326	26–591

^*^Data are from a single study

*ERA * Enthesitis-related arthritis, *JIA*   Juvenile idiopathic arthritis, *nRCT*   Nonrandomized controlled trial, *nsJIA   *Nonsystemic juvenile idiopathic arthritis, *PA*   Polyarticular arthritis, *RCT*   Randomized controlled trial, *SLR*   Systematic literate review, *sJIA*   Systemic juvenile idiopathic arthritis, *TNFi*   Tumor necrosis factor inhibitor

Of these 87 publications, 19 publications described a total of 13 RCTs, nRCTs, and open-label studies [13–16, 20, 34–47]. The studies mostly included cohorts with multiple JIA categories. The duration of randomized treatment phases ranged from 12 to 224 weeks, and treatment continued in the open-label extensions for up to 8 years. Quality assessment of the RCTs showed that all except 2 trials had a low overall risk of bias; the overall risk for the 2 exceptions was unclear (Supplementary Table 8). Quality assessment of the nRCTs showed that they all had a score of “fair” (Supplementary Table 9).

Of the 87 publications, 68 described observational studies spanning across 12 named registries and several unnamed data sources. The following numbers of publications were included on investigations of each TNFi: adalimumab, 23 [19]; etanercept, 56 [19]; golimumab, 5; and infliximab, 16 [14, 16]. Quality assessments of the observational studies showed that all except 1 study had a score of “fair” or better (Supplementary Table 10).

### Summary of efficacy outcomes

Across the RCTs, nRCTs, and open-label studies, the following percentages of patients achieved JIA-ACR responses at Week 12 after initiation of adalimumab (either as monotherapy or in combination with methotrexate): JIA-ACR30, 71–94%; JIA-ACR50, 68–90%; JIA-ACR70, 55–61%; and JIA-ACR90, 39–42% (Table 2). Corresponding proportions of patients achieving these responses at Week 12 after initiation of etanercept (mono- or combination therapy) were as follows: JIA-ACR30, 73–94%; JIA-ACR50, 53–78%; JIA-ACR70, 36–59%; and JIA-ACR90, 28% (Table 3). In the golimumab RCT, the following percentages of patients achieved JIA-ACR30, -ACR50, -ACR70, and -ACR90 at Week 16 after golimumab initiation: 89%, 79%, 66%, and 36%, respectively (Table 4). In the single study of infliximab (3 mg/kg in combination with methotrexate), JIA-ACR30, -ACR50, and -ACR70 responses were achieved at Week 14 in 64%, 50%, and 22% of patients, respectively (JIA-ACR90 was not reported) (Table 5).

**Table 2:** Primary outcomes in RCTs, nRCTs, and open-label studies of adalimumab

JIA categories	Patients, *n*	Time point	JIA-ACR 30/50/70/90, % of patients	Exposure, PY	SAE, *n*	SAE/100PY
NCT00048542 (3-part RCT) [15, 45]						
Multiple	ADA + MTX: 85	16 weeks lead-in	94/91/71/28	27.0	3	11.1
	ADA: 86		74/64/46/26	29.3	4	13.7
	PBO + MTX: 37	48 weeks randomized phase	38/38/27/27	15	1	6.7
	ADA + MTX: 38		63/63/63/42	18.3	0	0
	ADA: 30		57/53/47/30	14.4	0	0
	ADA + MTX: 71	104 weeks OLE	NR	127.4	7	5.5
	ADA: 57		NR	102.6	2	2.0
	ADA + /– MTX: 171	312 weeks	NR	592.8	75	12.7
NCT00775437 (Open-label study) [39]						
Multiple	ADA: 31	12 weeks	94/90/61/39	NR	X	X
	ADA: 30	24 weeks	90/83/73/37	NR	X	X
	ADA: 3	120 weeks	NR	45.1	5	11.1
NCT01166282 (RCT and OLE) [40]						
ERA	ADA: 31	12 weeks randomized phase	71/68/55/42	NR	1	NR
	ADA: 46	52 weeks OLE	> 80/ > 80/ > 75/ > 60	NR	5	NR

*ACR*   American College of Rheumatology, *ADA*   Adalimumab, *JIA*   Juvenile idiopathic arthritis, *MTX*   Methotrexate, *n*   Number of patients included in the analysis at the indicated timepoint, *NR*   Not reported, *nRCT*   Nonrandomized controlled trial, *OLE*   Open-label extension, *PBO*   Placebo, *PY*   Patient-years, *RCT*   Randomized controlled trial, *SAE*   Serious adverse event

**Table 3:** Primary outcomes in RCTs, nRCTs, and open-label studies of etanercept

JIA categories	Patients, n	Time point	JIA-ACR 30/50/70/90/100, % of patients	Exposure, PY	SAE, n	SAE/100PY
NCT00357903 (RCT with open-label wash-in, double-blind randomized treatment phase, and OLE) [13, 34–36]						
Multiple	ETN: 69	3 months	74/64/36/NR/NR	NR	NR	NR
	ETN: 69	1 year	NR	57	5	9.0
	ETN: 52	2 years	69/67/57/NR/NR	50	8	16.0
	ETN: 48	3 years	NR	45	9	20.0
	ETN: 42	4 years	NR	40	5	13.0
	ETN: 37	5 years	NR	36	2	6.0
	ETN: 34	6 years	NR	33	0	0
	ETN: 31	7 years	NR	29	4	14.0
	ETN: 69	LFU	83/77/61/41/18	318	39	12.0
PA onset	ETN: 30	24 months OLE	73/73/63/NR/NR	NR	NR	NR
TREAT (Randomized treat-to-target concept trial with treatment switch from placebo to active if target not reached) [37]						
PA	ETN + MTX + pred: 42	16 weeks	NR/NR/71/NR/NR	NR	NR	NR
	PBO + MTX + PBO: 43	16 weeks	NR/NR/44/NR/NR	NR	NR	NR
	ETN + MTX + pred: 30	24 weeks	NR/NR/40/NR/NR	NR	NR	NR
CLIPPER; NCT00962741 (Open-label study and OLE) [38, 42]						
Multiple	ETN: 127	12 weeks	86/78/59/28/22	NR	NR	NR
		96 weeks	84/84/79/55/46	215.1	16	7.4
ExOA	ETN: 60	12 weeks	90/NR/NR/NR/NR	NR	NR	NR
		96 weeks	88/88/83/55/48	103.6	2	1.9
ERA	ETN: 38	12 weeks	83/NR/NR/NR/NR	NR	NR	NR
		96 weeks	79/76/68/53/40	61.3	11	17.9
CLIPPER2; NCT01421069 (Long-term OLE of CLIPPER) [44]						
Multiple	ETN: 109	6 years	NR	524.4	32*	6.1*
ExOA	ETN: 55	6 years	NR	245.6	11*	4.5*
ERA	ETN: 31	6 years	NR	158.9	17*	10.7*
REMINDER (24-week, open-label treatment phase following a 24-week withdrawal-design RCT) [41]						
ERA	ETN: 41	24 weeks	93/93/80/56/46	18.2	1	5.5
BeSt for Kids-study; NTR1574 (randomized, treat-to-target concept trial) [43]						
Multiple	MTX/SSZ: 32	3 months	50/31/25/NR/NR	NR	2	NR
	MTX + pred: 32		53/38/19/NR/NR	NR	1	NR
	ETN + MTX: 30		73/53/47/NR/NR	NR	0	NR
EudraCT 2015–003,384–11 (RCT) [46]						
Multiple	ETN + MTX: 35	12 weeks	94/NR/NR/NR/NR	NR	NR	NR
	PBO + MTX: 33		61/NR/NR/NR/NR	NR	NR	NR
	ETN + MTX: 35	48 weeks	NR	NR	NR	NR
	PBO + MTX: 33		NR	NR	2	NR

^*^Treatment-emergent serious adverse events

*ACR*   American College of Rheumatology, *ERA*   Enthesitis-related arthritis, *ETN*   Etanercept, *ExOA*   Extended oligoarticular arthritis, *JIA*   Juvenile idiopathic arthritis, *LFU*   Last follow-up, *MTX*   Methotrexate, *n*   Number of patients included in the analysis at the indicated timepoint, *NR*   Not reported, *nRCT*   Nonrandomized controlled trial, *OLE*   Open-label extension, *PA*   polyarticular arthritis, *PBO*   Placebo, *pred*   Prednisolone, *PY*   Patient-years, *RCT*   Randomized controlled trial, *RF*   Rheumatoid factor, *SAE*   Serious adverse event, *SSZ*   Sulfasalazine

**Table 4:** Primary outcomes in RCTs, nRCTs, and open-label studies of golimumab

JIA categories	Patients, *n*	Time point	JIA-ACR 30/50/70/90, % of patients	Exposure, PY	SAE, *n*	SAE/100PY
GO KIDS; NCT01230827 (3-part RCT) [20]						
Multiple	GOL: 173	16 weeks OLE	89/79/66/36	53.7	8	16.8
	PBO: 76	48 weeks	NR	NR	10	32.5
	GOL: 78		53/51/47/39	NR	8	17.1
	PBO (to GOL): 76	96 weeks OLE	74/74/69/53	NR	7	10.4
	GOL: 78		69/69/65/49	NR	13	24.3
	GOL: 173	160 weeks	NR	325.6	39	18.1
NCT02277444 (1-arm, open-label study) [47]						
PA (RF −), PA (RF +), ERA, ExOA, PsA, sJIA	GOL: 127	28 weeks	84/80/70/47	NR	NR	NR
		52 weeks	76/74/65/49	NR	9	8.2

*ACR*   American College of Rheumatology, *ERA*   Enthesitis-related arthritis, *ExOA*   Extended oligoarticular arthritis, *GOL*   Golimumab, *JIA*   Juvenile idiopathic arthritis, *n*   Number of patients included in the analysis at the indicated timepoint, *NR*   Not reported, *nRCT*   Nonrandomized controlled trial, *OLE*   Open-label extension, *PA*   Polyarticular arthritis, *PBO*   Placebo, *PsA*   Psoriatic arthritis, *PY*   Patient-years, *RCT*   Randomized controlled trial, *RF*   Rheumatoid factor, *SAE*   Serious adverse event, *sJIA*   Systemic juvenile idiopathic arthritis

**Table 5:** Primary outcomes in RCTs, nRCTs, and open-label studies of infliximab

JIA categories	Patients, *n*	Time point	JIA-ACR 30/50/70/90, % of patients	Exposure, PY	SAE, *n* (%)	SAE/100PY
NCT00036374 (RCT with OLE) [14, 16]						
Multiple	PBO/IFX 6 mg/kg + MTX: 62	14 weeks	49/34/12/NR	NR	3 (5)	NR
	IFX 3 mg/kg + MTX: 60	14 weeks	64/50/22/NR	NR	NR	NR
	IFX 3 or 6 mg/kg + MTX: 112 (AEP)	16 weeks	73/NR/NR/NR	NR	NR	NR
	IFX 3 or 6 mg/kg + MTX: 112 (AEP)	52 weeks	NR/70/52/NR	NR	NR	NR
	IFX 3 or 6 mg/kg + MTX: 78 (OLE)	52 weeks	85/81/60/41	NR	NR	NR
	IFX 3 or 6 mg/kg + MTX: 78 (OLE)	204 weeks	44/40/33/24	NR	17 (21.8)	NR

*ACR*   American College of Rheumatology, *AEP*   all-evaluable population, *IFX*   *I*nfliximab, *JIA*   Juvenile idiopathic arthritis, *MTX*   Methotrexate, *n*   Number of patients included in the analysis at the indicated timepoint, *NR*   Not reported, *nRCT*   Nonrandomized controlled trial, OLE  Open-label extension, *PBO*   Placebo, *PY*   Patient-years, *RCT*   Randomized controlled trial, *SAE*   Serious adverse event

JIA-ACR30/50/70/90 rates were not reported in the observational studies of adalimumab or infliximab (Supplementary Table 11 and Supplementary Table 14, respectively). Across the observational studies of etanercept, the percentages of patients who achieved JIA-ACR30/50/70/90 after 3 months of treatment ranged from 72 to 98%, 54 to 86%, 28 to 66%, and 10 to 45%, respectively (Supplementary Table 12). In an observational study of subcutaneous golimumab, after 6 months of treatment, the percentages of patients who achieved these endpoints were 56%, 56%, 35%, and 21%, respectively (Supplementary Table 13). In a single-arm open-label study of intravenous golimumab, after 7 months of treatment, corresponding rates of JIA-ACR30/50/70/90 were 84%, 80%, 70%, and 47%, respectively (Table 4).

### Summary of safety outcomes

Across the RCTs, nRCTs, and open-label studies of adalimumab and etanercept in JIA, the incidence of SAEs ranged from 0 to 13.7 SAE/100PY and 0 to 20.0 SAE/100PY, respectively, across all time points for which data were available (Tables 2 and 3). In the single RCT of golimumab in which SAE data were available, the incidence of SAEs ranged from 10.4 to 24.3 SAE/100PY (Table 4). SAE incidence could not be obtained from the 2 publications describing the single study of infliximab (Table 5).

Across the observational studies of adalimumab and etanercept in JIA, the incidence of SAEs ranged from 0.8 to 11.0 SAE/100PY and 0.01 to 22.07 SAE/100PY, respectively, across all time points for which data were available (Supplementary Tables 11 and 12). The corresponding ranges after initiation of golimumab and infliximab were 2.7 to 5.32 SAE/100PY and 3.4 to 11.8 SAE/100PY, respectively (Supplementary Tables 13 and 14).

## Discussion

In this SLR, we identified many publications that assessed the use of TNFis in patients with JIA. The SLR included placebo-controlled randomized withdrawal trials and parallel-group trials, in addition to observational cohort studies. Efficacy estimates from these different designs are not comparable and should be viewed in light of the underlying study design. Overall, the data reported in these publications suggest that adalimumab, etanercept, golimumab, and infliximab are effective and well tolerated when used for the treatment of JIA. Because no publications describing studies of certolizumab pegol or TNFi biosimilars met the inclusion criteria of this SLR, the efficacy and safety of these agents in patients with JIA could not be examined. Most of the publications were of good quality, with a low risk of bias according to the RoB2 and NOS tools. However, of the RCTs identified, none were head-to-head studies of TNFis. In addition, most of the available evidence on TNFi treatment in JIA was derived from studies of etanercept (Table 1), reflecting in part the fact that this agent has the longest history of investigation in JIA.

Most of the studies that we identified included patient cohorts with multiple categories of JIA grouped under the functional concept of polyarticular-course JIA (namely, extended oligoarthritis, rheumatoid factor–positive or –negative polyarthritis, and/or systemic arthritis without active systemic symptoms in the prior 6 months). This JIA functional grouping was first introduced in the etanercept Phase 3 trial [13] and was adopted in subsequent TNFi Phase 3 trials by excluding patients with systemic onset JIA with recent systemic features. The approach, accepted by regulatory authorities such as the European Medicines Agency and U.S. Food and Drug Administration, was necessary because it was not feasible to conduct studies including patients with a single JIA category [48]. Studies that included populations with different categories of JIA provided little evidence of any difference in the efficacy and safety of TNFis when used in the different categories included within polyarticular-course JIA, although several reported reduced efficacy and increased discontinuation rates in patients with systemic JIA versus nonsystemic JIA. However, the latter differences should be interpreted with caution due to differences in disease severity/duration and treatment duration among the studies. A 2016 systematic review also provided some evidence of differences in responses to individual biologics by JIA category but noted the underrepresentation of some JIA categories in published studies [26].

The importance of transitional care for children with JIA has been demonstrated by findings that approximately 50% will continue to have active disease into adulthood, with further declines in physical health-related QoL and potential long-term disability [8–10]. In patients with JIA who continue to experience flares into adulthood, flares may preferentially impact previously inflamed joints, but patients are also at sustained risk for new joint accumulation [49]. A multifaceted clinical approach and optimal treatment are needed during the transition period. It should be emphasized that most data on the efficacy and safety of TNFis in the treatment of JIA were only available from children and adolescents. In fact, none of the publications of RCTs or nRCTs identified in the SLR included patients with JIA who were aged ≥ 18 years, and only 11 observational studies included such patients. Unfortunately, few of the latter observational studies reported evidence or discussed requirements relating to the transition from pediatric to adult care. Studies that include post-adolescent patients with long-term JIA are necessary to gain insight into the safety and effectiveness of TNFis when these agents are used through the transition from childhood to adulthood, including through periods of treatment discontinuation and/or switches. The challenges associated with patient follow-up through this transition period may explain, at least in part, the small number of studies that include such patients.

In addition to the absence of findings on treatment outcomes during the transition from pediatric to adult JIA care, several other important evidence gaps were identified in this SLR. Clinical evidence is lacking from TNFi RCTs, especially from head-to-head comparative studies. Additional data on clinical outcomes achieved with each TNFi across the different JIA categories would facilitate development of more specific treatment guidance, with the potential for improving outcomes. Most available evidence is derived from observational studies based on large patient registries, which have contributed essential information about the real-world safety, effectiveness, and tolerability of TNFi treatments developed within the context of clinical trials. For example, current evidence from observational studies indicates that infections are the most common adverse events and SAEs reported with the use of TNFis and other bDMARDs [50, 51]. Finally, in this SLR, we also found a considerable difference in the quantity of evidence available for each TNFi, with the more recently studied agents having the least information on their use. This gap is primarily attributable to differences in the development timelines of the TNFis included for review.

Only one infliximab study, which evaluated doses of 3 or 6 mg/kg, reported JIA-ACR response rates. For some indications, patients may benefit from higher doses of infliximab (e.g., up to 10 mg/kg every 8 weeks), and higher doses are sometimes used in clinical practice [52, 53]. However, no studies included in this SLR evaluated efficacy of doses of infliximab > 6 mg/kg.

This study builds on the findings of several prior systematic reviews and meta-analyses of efficacy and/or safety of bDMARDs in JIA that have been published between 2013 and 2020 [21–27]. For example, a 2016 meta-analysis of randomized withdrawal trials of biological agents in polyarticular JIA reported that the included biologics (abatacept, adalimumab, anakinra, etanercept, and tocilizumab) were similarly effective and safe compared with placebo [23]. The results of a 2020 meta-analysis of randomized controlled trials of biological agents in JIA also supported a net benefit in favor of biologic agents in the short term [27]. Given the need for long-term effectiveness data [22], it is important to continue to review emerging data from registries and long-term extension studies.

The present analysis has some specific limitations beyond those generally associated with the conduct of an SLR, such as the restriction to English-language publications and specific congresses and databases included in the search. The main limitation was the heterogeneity of the studies described in the publications identified, which prevents direct comparisons (such as meta-analysis) of evidence from clinical trials or observational studies. This heterogeneity arises largely from differences in the following features: 1) study design/methodology (e.g., outcomes, time of assessment/follow-up); 2) treatment arms (e.g., dosing, concomitant medications, treatment duration); and 3) patient populations (e.g., JIA of varying categories, duration, and severity; DMARD/biologic/TNFi-naïve, -intolerant, or -refractory). Another limitation of our analysis is the lack of a formal comparison of the patient populations across the different studies in terms of baseline disease severity. In addition, the exclusion of treatment arms containing fewer than 30 patients, implemented to increase the robustness of the data, is also considered a limitation. Moreover, a detailed description of SAEs reported in the included studies was beyond the scope of our SLR but may be worthy of additional analysis in future research. Finally, we note that the SLR was conducted according to PRISMA 2009 guidelines [28], which were current at the time the literature searches were conducted; however, updated PRISMA recommendations have recently been published [54].

In conclusion, the published evidence suggests that adalimumab, etanercept, golimumab, and infliximab are effective and well-tolerated treatments for JIA. This SLR could serve as the basis for a dedicated meta-analysis of the efficacy and safety of TNFis in JIA, as well as a future SLR dedicated to functional and QoL outcomes that would provide more precise guidance for the optimal use of TNFis in JIA.

## Supplementary Information

**Additional file 1.**

## Data Availability

Not applicable.

## Abbreviations

ACR: American College of Rheumatology
JIA: Juvenile idiopathic arthritis
NOS: Newcastle–Ottawa Scale
PRISMA: Preferred Reporting Items for Systematic Reviews and Meta-Analyses
QoL: Quality of life
RCT: Randomized controlled trials
SAE: Serious adverse event
SLR: Systematic literature review
