# Supplementary material for: Efficacy and safety of TNF inhibitors in the treatment of juvenile idiopathic arthritis: a systematic literature review
Source: Pediatr Rheumatol Online J. 2023 Feb 24;21:20. doi: 10.1186/s12969-023-00798-8 (PMC9951426; doi:10.1186/s12969-023-00798-8)
Supplement: Supplementary file 1 — Additional file 1. [file 12969_2023_798_MOESM1_ESM.docx]

**Safety and efficacy of TNF inhibitors in the treatment of juvenile idiopathic arthritis: a systematic literature review**

**Additional file 1 for Horneff et al. (2021)**

**Supplementary Table 1.** PRISMA checklist

| **Section and topic** | **Item** | **Checklist item** | **Page reported** |
| --- | --- | --- | --- |
| **Title** | | | |
| *Title* | 1 | Identify the report as a systematic review. | 1 |
| **Abstract** | | | |
| *Abstract* | 2 | See the PRISMA 2020 for Abstracts checklist. | 4 |
| **Introduction** | | | |
| *Rationale* | 3 | Describe the rationale for the review in the context of existing knowledge. | 6 |
| *Objectives* | 4 | Provide an explicit statement of the objective(s) or question(s) the review addresses. | 6 |
| **Methods** | | | |
| *Eligibility* | 5 | Specify the inclusion and exclusion criteria for the review and how studies were grouped for the syntheses. | 6, 7 |
| *Information sources* | 6 | Specify all databases, registers, websites, organizations, reference lists, and other sources searched or consulted to identify studies. Specify the date when each source was last searched or consulted. | 6, 7 |
| *Search strategy* | 7 | Present the full search strategies for all databases, registers and websites, including any filters and limits used. | Supplementary Tables 3–5 |
| *Selection process* | 8 | Specify the methods used to decide whether a study met the inclusion criteria of the review, including how many reviewers screened each record and each report retrieved, whether they worked independently, and if applicable, details of automation tools used in the process. | 7, 8 |
| *Data collection process* | 9 | Specify the methods used to collect data from reports, including how many reviewers collected data from each report, whether they worked independently, any processes for obtaining or confirming data from study investigators, and if applicable, details of automation tools used in the process. | 7, 8 |
| *Data items* | 10a | List and define all outcomes for which data were sought. Specify whether all results that were compatible with each outcome domain in each study were sought (e.g., for all measures, time points, analyses), and if not, the methods used to decide which results to collect. | 8, 9 |
|  | 10b | List and define all other variables for which data were sought (e.g., participant and intervention characteristics, funding sources). Describe any assumptions made about any missing or unclear information. | 8, 9 |
| *Study risk of bias assessment* | 11 | Specify the methods used to assess risk of bias in the included studies, including details of the tool(s) used, how many reviewers assessed each study and whether they worked independently, and if applicable, details of automation tools used in the process. | 8 |
| *Effect measures* | 12 | Specify for each outcome the effect measure(s) (e.g., risk ratio, mean difference) used in the synthesis or presentation of results. | 8, 9 |
| *Synthesis methods* | 13a | Describe the processes used to decide which studies were eligible for each synthesis (e.g., tabulating the study intervention characteristics and comparing against the planned groups for each synthesis [item #5]). | 7, 8 |
|  | 13b | Describe any methods required to prepare the data for presentation or synthesis, such as handling of missing summary statistics, or data conversions. | 8, 9 |
|  | 13c | Describe any methods used to tabulate or visually display results of individual studies and syntheses. | 8, 9 |
|  | 13d | Describe any methods used to synthesize results and provide a rationale for the choice(s). If meta-analysis was performed, describe the model(s), method(s) to identify the presence and extent of statistical heterogeneity, and software package(s) used. |  |
|  | 13e | Describe any methods used to explore possible causes of heterogeneity among study results (e.g., subgroup analysis, meta-regression). |  |
|  | 13f | Describe any sensitivity analyses conducted to assess robustness of the synthesized results. |  |
| *Reporting bias assessment* | 14 | Describe any methods used to assess risk of bias due to missing results in a synthesis (arising from reporting biases). | 8 |
| *Certainty assessment* | 15 | Describe any methods used to assess certainty (or confidence) in the body of evidence for an outcome. |  |
| **Results** | | | |
| *Study selection* | 16a | Describe the results of the search and selection process, from the number of records identified in the search to the number of studies included in the review, ideally using a flow diagram. | 9, Figure 1 |
|  | 16b | Cite studies that might appear to meet the inclusion criteria, but which were excluded, and explain why they were excluded. | Figure 1 |
| *Study characteristics* | 17 | Cite each included study and present its characteristics. | Supplementary Table 7 |
| *Risk of bias in studies* | 18 | Present assessments of risk of bias for each included study. | Supplementary Tables 8–10 |
| *Results of individual studies* | 19 | For all outcomes, present, for each study: (a) summary statistics for each group (where appropriate) and (b) an effect estimate and its precision (e.g., confidence/credible interval), ideally using structured tables or plots. | Tables 2–5,  Supplementary Tables 11–14 |
| *Results of syntheses* |  | For each synthesis, briefly summarize the characteristics and risk of bias among contributing studies. | Supplementary Tables 8–10 |
|  | 20a | Present results of all statistical syntheses conducted. If meta-analysis was done, present for each the summary estimate and its precision (e.g., confidence/credible interval) and measures of statistical heterogeneity. If comparing groups, describe the direction of the effect. |  |
|  | 20b | Present results of all investigations of possible causes of heterogeneity among study results. |  |
|  | 20c | Present results of all sensitivity analyses conducted to assess the robustness of the synthesized results. |  |
| *Reporting biases* | 21 | Present assessments of risk of bias due to missing results (arising from reporting biases) for each synthesis assessed. | Supplementary Tables 8–10 |
| *Certainty of evidence* | 22 | Present assessments of certainty (or confidence) in the body of evidence for each outcome assessed. |  |
| **Discussion** | | | |
| *Discussion* | 23a | Provide a general interpretation of the results in the context of other evidence. | 12–15 |
|  | 23b | Discuss any limitations of the evidence included in the review. | 14, 15 |
|  | 23c | Discuss any limitations of the review processes used. | 14 |
|  | 23d | Discuss implications of the results for practice, policy, and future research. | 12–15 |
| **Other information** | | | |
| *Registration and protocol* | 24a | Provide registration information for the review, including register name and registration number, or state that the review was not registered. | 6, 19, 20 |
|  | 24b | Indicate where the review protocol can be accessed, or state that a protocol was not prepared. | 6, 19, 20 |
|  | 24c | Describe and explain any amendments to information provided at registration or in the protocol. |  |
| *Support* | 25 | Describe sources of financial or non-financial support for the review, and the role of the funders or sponsors in the review. | 2, 3 |
| *Competing interests* | 26 | Declare any competing interests of review authors. | 2, 3 |
| *Availability of data, code and other materials* | 27 | Report which of the following are publicly available and where they can be found: template data collection forms; data extracted from included studies; data used for all analyses; analytic code; any other materials used in the review. |  |

**Supplementary Table 2.** PICOS criteria applied to the online literature searches

| **Criterion** | **Definitions applied to searches** |
| --- | --- |
| Population | Children, adolescents, and adults with JIA categorized as polyarthritis (rheumatoid factor positive or negative), extended oligoarthritis, psoriatic arthritis, enthesitis-related arthritis, or systemic arthritis. |
| Intervention and comparator | Monotherapy or combination therapy (with conventional DMARD or other agent) with adalimumab, certolizumab pegol, etanercept, golimumab, or infliximab (including Benepali®, Eticovo®, Erelzi®, Amgevita®, Hadlima®, Hulio®, Hyrimoz®, Imraldi®, Abrilada®, Inflectra®, Renflexis®, Ixifi®, and Avsola® biosimilars). |
| Outcomes | *Efficacy/Effectiveness*  JIA-ACR score/ACR pediatric score, JADAS, inactive disease, remission (6 months of inactive disease), C-reactive protein, erythrocyte sedimentation rate, active joint count, swollen joint count, joints with limitation of motion, Physician’s Global Assessment, Patient/Parent Global Assessment, treatment duration, drug survival, discontinuation due to inefficacy, and discontinuation due to remission.  *Safety*  Exposure-adjusted AEs (grade 3+), serious AEs, treatment-emergent AEs, treatment-related AEs, discontinuation due to AE, incidence of anti-drug antibodies, immunogenicity, death, and mortality.  *Quality of life*  Quality-adjusted life years, disability-adjusted life years, CHQ, EQ-5D, PedsQL, PQRL, RAND-36, SF36, visual analog scales, and other tools. |
| Study types | Clinical trials and observational studies. |
|  | |

ACR = American College of Rheumatology; AE = adverse event; CHQ = Child Health Questionnaire™; DMARD = disease-modifying anti-rheumatic drug; EQ-5D = EuroQol-5 Dimension; JADAS = Juvenile Arthritis Disease Activity Score; JIA = juvenile idiopathic arthritis; PedsQL = Pediatric Quality of Life Inventory™; PICOS = population, intervention, comparator, outcomes, and study types; PRQL = Pediatric Rheumatology Quality of Life Scale; RAND-36 = RAND 36-Item Health Survey; SF36 = Short Form Health Survey Questionnaire.**Supplementary Table 3.** Search strategy for Embase®

| **Step** | **Search string** | **Hits** |
| --- | --- | --- |
| 1 | exp juvenile rheumatoid arthritis/ | 20,890 |
| 2 | ((Juvenile adj2 arthritis) or (still* adj2 disease) or JIA or JRA or sJIA or soJIA).mp | 35,909 |
| 3 | 1 or 2 | 35,909 |
| 4 | (Etanercept or Enbrel or ETA or ETN).mp. | 54,545 |
| 5 | (Infliximab or remicade or ABP 710 or ABP-710 or BOW-015 or BOW015 or CT-P-13 or CT-P13 or GP 1111 or GP-1111 or PF-06438179 or TA-650).mp | 50,569 |
| 6 | (Adalimumab or humira or hum?ra).mp | 33,615 |
| 7 | (Certolizumab pegol or Cimzia or CDP 870 or CDP-870 or CDP870 or PHA-738144).mp | 6,432 |
| 8 | (Golimumab or Simponi or CNTO 148).mp | 6,750 |
| 9 | (Benepali or Brenzys or Eticovo or SB4 or etanercept-ykro or Erelzi or GP-2015 or GP2015 or etanercept-szzs or Amjevita or Amgevita or ABP501 or Hulio or Hyrimoz or adalimumab-adaz or GP 2017 or Imraldi or SB5 or Abrilada or Hadlima or adalimumab- bwwd or inflectra or renflexis or Ixifi or avsola or infliximab-dyyb or infliximab-abda or infliximab- qbtx or infliximab- axxq).mp | 895 |
| 10 | (anti-TNF* or TNFi* or (TNF* adj blocker) or (TNF* adj inhibit*)).ti | 7,526 |
| 11 | Or/4-10 | 10,0718 |
| 12 | 3 and 11 | 4,283 |
| 13 | (editorial or comment* or letter or note or case series or case study or case studies or case report or conference abstract).pt. or (editorial/ or letter/ or case study/ or case report/ or note/) | 831,4437 |
| 14 | 12 not 13 | 2261 |
| 15 | (animal$ not human$).sh,hw. | 4,328,453 |
| 16 | 14 not 15 | 2,253 |
| 17 | limit 16 to English language | 2,019 |
| 18 | Remove duplicates from 17 | 1,985 |

**Supplementary Table 4.** Search strategy for MEDLINE®

| **Step** | **Search string** | **Hits** |
| --- | --- | --- |
| 1 | exp Arthritis, Juvenile/ | 10,287 |
| 2 | ((Juvenile adj2 arthritis) or (still* adj2 disease) or JIA or JRA or sJIA or soJIA).mp | 21,785 |
| 3 | 1 or 2 | 21,785 |
| 4 | (Etanercept or Enbrel or ETA or ETN).mp. | 29,275 |
| 5 | (Adalimumab or humira or hum?ra).mp | 8,589 |
| 6 | (Certolizumab pegol or Cimzia or CDP 870 or CDP-870 or CDP870 or PHA-738144).mp | 1,000 |
| 7 | (Infliximab or remicade or ABP 710 or ABP-710 or BOW-015 or BOW015 or CT-P-13 or CT-P13 or GP 1111 or GP-1111 or PF-06438179 or TA-650).mp | 14,624 |
| 8 | (Golimumab or Simponi or CNTO 148).mp | 1,187 |
| 9 | (Benepali or Brenzys or Eticovo or SB4 or etanercept-ykro or Erelzi or GP-2015 or GP2015 or etanercept-szzs or Amjevita or Amgevita or ABP501 or Hulio or Hyrimoz or adalimumab-adaz or GP 2017 or Imraldi or SB5 or Abrilada or Hadlima or adalimumab- bwwd or inflectra or renflexis or Ixifi or avsola or infliximab-dyyb or infliximab-abda or infliximab- qbtx or infliximab- axxq).mp. | 373 |
| 10 | (anti-TNF* or TNFi* or (TNF* adj blocker) or (TNF* adj inhibit*)).ti. | 3,229 |
| 11 | Or/4-10 | 46,325 |
| 12 | 3 and 11 | 988 |
| 13 | (editorial or comment* or letter or note or case series or case study or case studies or case report).pt. or (editorial/ or letter/ or case study/ or case report/ or note/) | 3,691,480 |
| 14 | 12 not 13 | 705 |
| 15 | (animals not (humans and animals)).sh. | 4,645,503 |
| 16 | 14 not 15 | 702 |
| 17 | limit 16 to English language | 621 |
| 18 | Remove duplicates from 17 | 616 |

**Supplementary Table 5.** Search strategy for PubMed®

| **Step** | **Search string** | **Hits** |
| --- | --- | --- |
| 1 | arthritis, juvenile idiopathic[MeSH Terms] | 10,284 |
| 2 | (juvenile[Text Word]) AND arthritis[Text Word] | 13,537 |
| 3 | (JIA[Text Word]) OR JRA[Text Word] OR soJIA [Text Word] or sJIA [Text Word] or still’s disease [Text Word] | 7,423 |
| 4 | #1 or #2 or #3 | 15,761 |
| 5 | (Etanercept or Enbrel or ETA or ETN) | 28,185 |
| 6 | (Adalimumab or humira) | 8,176 |
| 7 | (Certolizumab pegol or Cimzia or CDP 870 or CDP-870 or CDP870 or PHA-738144) | 994 |
| 8 | (Infliximab or remicade or ABP 710 or ABP-710 or BOW-015 or BOW015 or CT-P-13 or CT-P13 or GP 1111 or GP-1111 or PF-06438179 or TA-650) | 14,597 |
| 9 | (Golimumab or Simponi or CNTO 148) | 1,186 |
| 10 | (Benepali or Brenzys or Eticovo or SB4 or etanercept-ykro or Erelzi or GP-2015 or GP2015 or etanercept-szzs or Amjevita or Amgevita or ABP501 or Helio or Hyrimoz or adalimumab-adaz or GP 2017 or Imraldi or SB5 or Abrilada or Hadlima or adalimumab-bwwd) | 16,870 |
| 11 | (anti-TNF* or TNFi* or TNF* blocker or TNF* inhibit*) | 89,263 |
| 12 | #5 or #6 or #7 or #8 or #9 or #10 or #11 | 129,277 |
| 13 | #4 and #12 | 1,129 |
| 14 | (editorial[Publication Type] OR comment[Publication Type] OR comments[Publication Type] OR commentary [Publication Type] letter[Publication Type] OR note[Publication Type] OR case series[Publication Type] OR case study[Publication Type] OR case studies[Publication Type] OR case reports[Publication Type] OR congress [Publication Type]) | 2,590,694 |
| 15 | (animal[Title] or animals [Title] OR murine[Title] OR mouse[Title] OR mice[Title] OR rodent* [Title] or swine[Title] OR pig*[Title] OR porcine*[Title] OR rat[Title] or rats [Title] OR monkey*[Title] OR dog*[Title] OR canine*[Title] OR cat[Title] OR feline*[Title]) | 1,982,723 |
| 16 | #14 or #15 | 4,541,239 |
| 17 | #13 not #16 | 867 |
| 18 | Limit #17 to English | 784 |

**Supplementary Table 6.** Inclusion and exclusion criteria

| **Inclusion criteria** | |
| --- | --- |
| Population | - Patients with JIA (children, adolescents and adults), including - polyarthritis (RF+ or RF–) - extended oligoarthritis - psoriatic arthritis - enthesitis-related arthritis - systemic arthritis |
| Intervention* | - Adalimumab - Certolizumab pegol - Etanercept - Golimumab - Infliximab - Biosimilars: Benepali, Eticovo, Erelzi, Amgevita, Hadlima, Hulio, Hyrimoz, Imraldi, Abrilada, Inflectra, Renflexis, Ixifi, Avsola |
| Outcomes | - Efficacy/Effectiveness:   - JIA-ACR score/ACR pediatric score   - Juvenile arthritis disease activity score   - Percentage of patients in inactive disease, remission (on/off drug)   - C-reactive protein and erythrocyte sedimentation rates   - Active joint count, swollen joint count, tender joint count   - Definition of motion, joints with limitation of motion   - Physician’s Global Assessment, patient/Parent Global Assessment   - Duration of treatment   - Drug survival, discontinuation due to inefficacy, discontinuation due to remission - Safety   - Exposure-adjusted AEs (Grade 3+)   - SAEs   - TEAEs   - TRAEs   - Anti-drug antibodies, immunogenicity   - Death, mortality   - Discontinuation due to AE - Quality of life   - QALY   - DALY   - CHQ   - PedsQL   - SF36   - RAND-36   - EuroQoL   - PRQL   - Visual analog scales (and other identified HRQoL tools) |
| Study design | - Clinical trials - Observational studies |
| **Exclusion criteria** | |
| Language | - Studies not published in English |
| Population | - Studies with <30 patients - Studies focused specifically on JIA patients with uveitis |
| Study type | - Preclinical and case studies, case series, cell-based or gene expression studies (including biomarker/predictor studies), methodological studies, modeling studies, notes, commentaries, editorials, opinions, economic model studies, meta-analyses, reviews^†^ |
| Intervention | - Studies reporting non-TNFi treatment or where outcomes for several anti-TNF treatments are mixed/undefined - Studies in patients who previously received TNFi but currently in remission/not undergoing treatment (i.e., discontinuation studies) - Studies focused on the switching of drugs for non-medical reasons |
|  | |

*Monotherapy or in combination with a disease-modifying anti-rheumatic drug or no comparator

^†^Reviews were excluded but reference lists for relevant reviews were screened for primary sources

RF = rheumatoid factor; TNFi = tumor necrosis factor inhibitor.

**Supplementary Table 7.** Summary of publications included after online literatures searches and screening processes

| Source | Study name/ registry | Country | Study design | Patient population | JIA category | Intervention |  |
| --- | --- | --- | --- | --- | --- | --- | --- |
| **RCT** | | | | | | | |
| Alexeeva et al, 2021 (1) | NR | Russia | Randomized, double-blind, placebo-controlled | Biologic-naïve and not receiving DMARD | Multiple | ETN + MTX |  |
| Brunner et al, 2018 (2) | GO KIDS | Multinational | Phase 3, 3-part, randomized, double-blind, placebo-controlled | 80% biologic-naïve; MTX-refractory | Multiple | GOL |  |
| Burgos-Vargas et al, 2015 (3) | NR | Multinational | Phase 3, randomized, double-blind, placebo-controlled | Biologic-naïve; DMARD-refractory or -intolerant | ERA | ADA |  |
| Hissink Muller et al, 2017 (4) | BeSt for Kids-study | Netherlands | 3-armed, randomized, single-blind | Biologic- and DMARD-naïve | nsJIA | MTX/SSZ MTX + pred ETN + MTX |  |
| Horneff et al, 2015 (5) | REMINDER | Germany | Phase 1, 2-part, open-label run-in, randomized | Biologic-naïve; DMARD -refractory or -intolerant | ERA | ETN |  |
| Lovell et al, 2000 (6) | NR | US | Phase 3, 2-part, open-label run-in, randomized | Biologic-naïve; DMARD-refractory or -intolerant | Multiple | ETN |  |
| Lovell et al, 2003 (7) | NR | US | Phase 3, open-label extension | Biologic-naïve; DMARD-refractory or -intolerant | Multiple | ETN |  |
| Lovell et al, 2006 (8) | NR | US | Phase 3, open-label extension | Biologic-naïve; DMARD-refractory or -intolerant | Multiple | ETN |  |
| Lovell et al, 2008 (9) | NR | US | Phase 3, open-label extension | Biologic-naïve; DMARD-refractory or -intolerant | Multiple | ETN |  |
| Lovell et al, 2008 (10) | NR | Multinational | 3-part, randomized, double-blind, placebo-controlled | Biologic-naïve; MTX-naïve or MTX -refractory | Multiple | ADA  ADA + MTX |  |
| Lovell et al, 2020 (11) | NR | Multinational | 3-part, randomized, double-blind, placebo-controlled | Biologic-naïve; MTX-naïve or MTX -refractory | Multiple | ADA +/− MTX |  |
| Ruperto et al, 2007 (12) | NR | Multinational | Phase 3, randomized, double-blind | Biologic-naïve; DMARD-refractory | Multiple | INF + MTX |  |
| Ruperto et al, 2010 (13) | NR | Multinational | Phase 3, open-label extension | Biologic-naïve; DMARD-refractory | Multiple | INF + MTX |  |
| Wallace et al, 2012 (14) | TREAT | US | Randomized, double-blind, placebo-controlled | Biologic-naïve; DMARD-naïve or -experienced | PA | ETN + MTX + pred PBO + MTX |  |
| **nRCT** | | | | | | | |
| Constantin et al, 2016 (15) | CLIPPER | Multinational | Phase 3b, non-randomized, open-label | Biologic-naïve; DMARD-refractory or -intolerant | Multiple, ERA, ExOA, PsA | ETN |  |
| Foeldvari et al, 2019 (16) | CLIPPER2 | Multinational | Phase 3b, non-randomized, open-label | Biologic-naïve; DMARD-refractory or -intolerant | Multiple, ERA, ExOA, PsA | ETN |  |
| Horneff et al, 2014 (17) | CLIPPER | Multinational | Phase 3b, non-randomized, open-label | Biologic-naïve; DMARD-refractory or -intolerant | Multiple, ERA, ExOA, PsA | ETN |  |
| Kingsbury et al, 2014 (18) | NR | EU, US | Phase 3b, non-randomized, open-label | Biologic-naïve; DMARD-refractory; moderate to severe active JIA | Multiple, ExOA, PA (RF– and RF+), sJIA, undiff | ADA |  |
| Ruperto et al, 2021 (19) | NR | Multinational | Phase 3, open-label, single-arm | Mixed | Multiple | GOL |  |
| **Observational** | | | | | | | |
| Aeschlimann et al, 2014 (20) | NR | Switzerland | Retrospective | Biologic- experienced; DMARD-refractory | Multiple | INF |  |
| Armaroli et al, 2020 (21) | BiKeR | Germany | Retrospective | Mixed | Multiple | ETN |  |
| Alexeeva et al, 2017 (22) | NR | Russia | Prospective | Biologic (ETN)- naïve and/or other biologic- experienced | Multiple, POA, PA (RF−) | ETN |  |
| Alexeeva et al, 2019 (23) | NR | Russia | Post hoc analysis of prospective observational study | Biologic (ETN)- naïve | Multiple | ETN + MTX  MTX |  |
| Bader-Meunier et al, 2019 (24) | NR | France | Prospective | Biologic- experienced | Multiple | ETN |  |
| Becker et al, 2017 (25) | BIKER | Germany | Prospective | Biologic-naïve; DMARD- experienced and -naïve; MTX-naïve (MTX control cohort only) | Multiple | ETN or MTX or ETN + MTX or ADA or ADA + MTX |  |
| Beukelman et al, 2016 (26) | NR | US | Retrospective | Biologic-naïve; MTX-naïve | Multiple, sJIA | MTX, ADA, CPZ, ETN, GOL, INF |  |
| Brunelli et al, 2020 (27) | NR | Brazil | Prospective | Biologic- experienced; DMARD- experienced | Multiple | ADA |  |
| Brunner et al, 2020 (28) | STRIVE | Multinational | Prospective | Biologic-naïve (MTX mono cohort only); ADA mono cohort included MTX-intolerant or  -refractory | Multiple | MTX, ADA, ADA + MTX, ADA ± MTX |  |
| Cabrera et al, 2019 (29) | JIRcohorte | Multinational | Retrospective | Biologic- experienced and -naïve | Multiple | ADA, ANK, CAN, ETN, GOL, INF, TOC |  |
| Choi et al, 2018 (30) | NR | Korea | Retrospective | Biologic- experienced and -naïve | PA | ETN |  |
| Davies et al, 2015 (31) | BSPAR-ETN | UK | Prospective | Biologic-naïve (MTX cohort); MTX-refractory (ETN cohort) | Multiple | ETN  MTX  ETN + MTX |  |
| Dumaine et al, 2020 (32) | JIRcohorte | Multinational | Retrospective | Biologic- experienced and -naïve | Multiple | ADA, ANK, ABT, CAN, ETN, INF, TOC |  |
| Favalli et al, 2017 (33) | NR | Italy | Retrospective | Biologic-naïve; DMARD- experienced and -naïve | Multiple | ADA  ETN  INF |  |
| Feger et al, 2019 (34) | NR | USA | Prospective | Biologic- experienced and -naïve; DMARD- experienced and -naïve | PA | ETN |  |
| Finetti et al, 2018 (35) | ABIRISK/ Pharmachild | Multinational | Prospective | Biologic-naïve, NR; DMARD, NR | Multiple | ADA  ETN  TOC |  |
| Geikowski et al, 2014 (36) | BIKER | Germany | Prospective | Biologic-naïve; MTX- experienced | Multiple | ETN |  |
| Gerloni et al, 2008 (37) | NR | Italy | Prospective | Biologic-naïve; DMARD-refractory or -intolerant | Multiple | ETN  INF |  |
| Giannini et al, 2009 (38) | NR | US, Canada | Prospective, phase 4 | Biologic- experienced and -naïve; DMARD- experienced and -naïve | PA, sJIA | ETN  MTX  ETN + MTX |  |
| Glazyrina et al, 2019 (39) | NR | Russia | Prospective | Biologic-naïve; MTX -refractory | Multiple | ETN |  |
| Goettel et al, 2021 (40) | NR | US | Retrospective | Mixed | Mixed | ETN  ADA |  |
| Haapasaari et al, 2002 (41) | NR | Finland | Retrospective | Biologic-naïve; MTX -refractory | Multiple | ETN |  |
| Halbig et al, 2009 (42) | BIKER | Austria, Germany | Prospective | Biologic-naïve; MTX -refractory | Multiple | ETN |  |
| Horneff et al, 2004 (43) | BIKER | Austria, Germany | Prospective | Biologic-naïve; DMARD-refractory | Multiple | ETN |  |
| Horneff et al, 2009 (44) | BIKER | Germany | Prospective | Biologic-naïve; DMARD-refractory | Multiple | ETN ETN + MTX |  |
| Horneff et al, 2016 (45) | BIKER | Germany | Prospective | Biologic- experienced and -naïve | PA | ETN  ADA |  |
| Horneff et al, 2017 (46) | BIKER | Germany | Prospective | Biologic- experienced; DMARD- experienced and -naïve | sJIA | ETN  TOC  IL-1i |  |
| Horneff et al, 2019 (47) | BIKER | Germany | Prospective | Biologic- experienced and -naïve; DMARD- experienced and -naïve | Multiple | ADA  MTX |  |
| Horneff et al, 2020 (48) | BiKeR | Germany | Prospective | Biologic-naïve | Multiple | GOL  MTX |  |
| Kearsley-Fleet et al, 2016 (49) | BSPAR-ETN | UK | Retrospective | Biologic-naïve; DMARD- experienced | Multiple | ETN |  |
| Kimura et al, 2005 (50) | NR | US | Retrospective | Biologic-naïve; DMARD- experienced | sJIA | ETN |  |
| Klein et al, 2019 (51) | BIKER | Germany | Prospective | Biologic- experienced and -naïve; DMARD- experienced and -naïve | Multiple | ADA  ADA + MTX |  |
| Klein et al, 2019 (52) | BIKER | Germany | Prospective | Biologic- experienced and -naïve; DMARD- experienced and -naïve | sJIA | ETN, TOC, ANK, CAN |  |
| Klein et al, 2020 (53) | BIKER | Germany | Prospective | Biologic- experienced and -naïve; DMARD- experienced and -naïve | PA | ETN, ADA, GOL, INF, TOC, ABT, MTX |  |
| Klein et al, 2020 (53) | BIKER | Germany | Prospective | Biologic- experienced and -naïve; DMARD- experienced and -naïve | sJIA | ETN, TOC, ANK, CAN |  |
| Klotsche et al, 2014 (54) | BiKeR | Germany | Prospective | DMARD- experienced and -naïve | Multiple | ETN |  |
| Klotsche et al, 2016 (55) | BIKER/ JuMBO | Germany | Prospective | Biologic-naïve, DMARD- experienced and -naïve | Multiple | MTX  ETN  ADA |  |
| Klotsche et al, 2019 (56) | BIKER/ JuMBO | Germany | Prospective | Biologic-naïve | Multiple | ETN |  |
| Klotsche et al, 2020 (57) | BIKER/ JuMBO | Germany | Prospective | NR | NR | ETN |  |
| McErlane et al, 2013 (58) | BSRBR-RA | UK | Retrospective | Biologic-naïve, DMARD- experienced and -naïve | Multiple | ADA  ETN  INF |  |
| Minden et al, 2012 (59) | JuMBO | Germany | Prospective | Biologic-naïve | Multiple, ERA, ExOA, PsA, PA (RF−), PA (RF+), sJIA, other JIA | ETN |  |
| Nielsen et al, 2008 (60) | ANTARES | Italy | Retrospective | DMARD-refractory or -intolerant | Multiple | ETN |  |
| Otten et al, 2013 (61) | ABC | Netherlands | Prospective | Biologic-naïve and -experienced; DMARD-naïve and -experienced | Multiple, sJIA, nsJIA | ETN  ADA |  |
| Otten et al, 2011 (62) | ABC | Netherlands | Prospective | Biologic-naïve; DMARD- experienced | Multiple, OA, PA, sJIA | ETN |  |
| Pastore et al, 2018 (63) | NR | Italy | Retrospective | Biologic- experienced and -naïve | Multiple | ETN |  |
| Prince et al, 2010 (64) | ABC | Netherlands | Prospective | Biologic-naïve, NR; DMARD- experienced | Multiple | ETN |  |
| Prince et al, 2009 (65) | ABC | Netherlands | Prospective | Biologic-naïve; MTX -refractory or -intolerant | Multiple | ETN |  |
| Quartier et al, 2003 (66) | NR | France | Prospective | DMARD- experienced; all MTX-refractory or -intolerant | Multiple | ETN |  |
| Romano et al, 2014 (67) | NR | Italy | Prospective | Biologic-naïve; DMARD-refractory or -intolerant | Multiple | ETN  INF  ADA |  |
| Ruperto et al, 2019 (68) | NR | Multinational | Prospective | MTX-refractory or -intolerant | PA | GOL |  |
| Russo et al, 2009 (69) | NR | Argentina | Prospective | Biologic-naïve; MTX -refractory | sJIA | ETN |  |
| Schmeling et al, 2014 (70) | BIKER | Germany | Prospective | Biologic- experienced and -naïve; DMARD - experienced (>95%) | Multiple | ADA |  |
| Sevcic et al, 2011 (71) | NIRPR | Hungary | Prospective | Biologic-naïve and -experienced; DMARD- experienced | Multiple | ETN |  |
| Solari et al, 2013 (72) | NR | Italy | Retrospective | Biologic-naïve; DMARD- experienced | Multiple | ETN |  |
| Southwood et al, 2011 (73) | BSPAR  (and BNDR) | UK | Prospective and retrospective | DMARD- experienced; MTX-intolerant or -refractory | Multiple | ETN |  |
| Su et al, 2017 (74) | NR | Taiwan | Retrospective | Biologic-naïve; MTX -intolerant or -refractory | Multiple | ETN |  |
| Takei et al, 2021 (75) | NR | Japan | Prospective | Mixed | Multiple | ADA |  |
| Tambralli et al, 2013 (76) | NR | US | Retrospective | Biologic- experienced and -naïve; DMARD- experienced and - naïve | Multiple | INF |  |
| Tarkiainen et al, 2015 (77) | NR | Finland | Prospective | Biologic- experienced and -naïve; DMARD- experienced and -naïve | Multiple | ETN  INF  ADA |  |
| Trachana et al, 2013 (78) | NR | Greece | Retrospective | Biologic- experienced and -naïve; DMARD -refractory | Multiple | ETN |  |
| Tynjälä et al, 2009 (79) | NR | Finland | Retrospective | Biologic-naïve, DMARD- experienced | Multiple | ETN  INF |  |
| Verazza et al, 2016 (80) | EtICA | Italy | Retrospective and cross-sectional | Biologic- experienced and -naïve, DMARD- experienced; whole population | Multiple | ETN |  |
| Verstegen et al, 2020 (81) | NR | NR | Retrospective | NR | NR | ETN  ADA |  |
| Windschall et al, 2015 (82) | BIKER | Germany | Prospective | Biologic- experienced and -naïve; DMARD- experienced and -naïve | ERA, PsA, ExOA, PA (RF−), PA (RF+) | ETN |  |
| Windschall et al, 2016 (83) | BIKER | Germany | Prospective | Biologic-naïve; MTX -refractory | PA | ETN |  |
| Zuber et al, 2011 (84) | Polish JIA registry | Poland | Retrospective | Biologic-naïve, NR; MTX-refractory or -intolerant | Multiple | ETN |  |

ABC = Arthritis and Biologicals in Children; ABIRISK = Anti-Biopharmaceutical Immunization = Prediction and Analysis of Clinical Relevance to Minimize the Risk; ADA = adalimumab; ANK = anakinra; ANTARES = anti-TNF therapy in rheumatoid arthritis registry; BeSt = Treatment Strategies in Rheumatoid Arthritis; BIKER = German Biologics in Pediatric Rheumatology; BNDR = Biologics and New Drugs Registry; BSPAR-ETN = British Society for Paediatric and Adolescent Rheumatology Etanercept Cohort Study; CAN = cankinumab; CLIPPER = Clinical Study In Paediatric Patients of Etanercept for Treatment of ERA, PsA, and Extended Oligoarthritis; DMARD = disease-modifying anti-rheumatic drug; ERA = enthesitis-related arthritis; EtICA = Etanercept in Italian Children with Arthritis; ETN = etanercept; EU = Europe; ExOA = extended oligoarticular arthritis; GOL = golimumab; IL-1i = interleukin-1 inhibitor; INF = infliximab; IQR = interquartile range; JIA = juvenile idiopathic arthritis; JuMBO = Juvenile arthritis MTX/Biologics long-term Observation; mono = monotherapy; MTX = methotrexate; NIRPR = National Institute of Rheumatology and Physiotherapy Registry; NR = not reported; nRCT = non-randomized controlled trial; nsJIA = non-systemic juvenile idiopathic arthritis; OA = oligoarticular arthritis; PA = polyarticular arthritis; PBO = placebo; POA = persistent oligoarticular arthritis; pred = prednisone; PsA = psoriatic arthritis; pts = patients; RCT = randomized controlled trial; RF = rheumatoid factor; SAE = serious adverse events; SD = standard deviation; sJIA = systemic juvenile idiopathic arthritis; SSZ = sulfasalazine; TOC = tocilizumab; TREAT = Trial of Early Aggressive Therapy; undiff = undifferentiated; UK = United Kingdom; US = United States.

**Supplementary Table 8.** Quality assessment of RCTs using ROB2

| **Source** | **Sequence generation** | **Allocation concealment** | **Blinding of participants, personnel** | **Blinding of outcome assessors** | **Incomplete outcome data** | **Selective outcome reporting** | **Overall** |
| --- | --- | --- | --- | --- | --- | --- | --- |
| Alexeeva et al, 2021 (1) | **+** | **+** | **+** | **+** | **+** | **+** | **+** |
| Wallace et al, 2012 (14) | **+** | **+** | **+** | **?** | **+** | **+** | **+** |
| Lovell et al, 2000, 2003, 2006, 2008 (6-9) | **+** | **+** | **+** | **+** | **+** | **+** | **+** |
| Horneff et al, 2015 (5) | **?** | **+** | **+** | **+** | **+** | **+** | **+** |

Key: + = low risk of bias; ? = unclear risk of bias.

ROB2 = Risk of Bias Tool version 2.

**Supplementary Table 9.** Quality assessment of nRCTs using Newcastle–Ottawa Scale

| **Source** | **Selection** | | | | **Comparability** | **Outcome** | | | **Total** |
| --- | --- | --- | --- | --- | --- | --- | --- | --- | --- |
|  | **Represent-ativeness of exposed cohort** | **Selection of non-exposed cohort** | **Ascertain-ment of exposure** | **Outcome of interest not present at start** | **Comparability of cohorts on basis of the design or analysis** | **Assess-ment of outcome** | **Follow-up long enough for outcomes to occur** | **Adequacy of follow-up of cohorts** |  |
| Horneff et al, 2014 (17)  Constantin et al, 2016 (15)  Foeldvari et al, 2019 (16) | 1 | 0 | 1 | 0 | 0 | 1 | 1 | 1 | 5 |
| Kingsbury et al, 2014 (18) | 1 | 0 | 1 | 0 | 0 | 1 | 1 | 1 | 5 |
| Ruperto et al, 2021 (19) | 1 | 0 | 1 | 0 | 0 | 1 | 1 | 1 | 5 |
|  | | | | | | | | | |

Interpretation of scores: 0–3 = poor, 4–5 = fair, ≥6 = good.

**Supplementary Table 10.** Quality assessment of observational studies using Newcastle–Ottawa Scale

| **Source** | **Selection** | | | | **Comparability** | **Outcome** | | | **Total** |
| --- | --- | --- | --- | --- | --- | --- | --- | --- | --- |
|  | **Representativeness of the exposed cohort** | **Selection of the non-exposed cohort** | **Ascertainment of exposure** | **Outcome of interest not present at start** | **Comparability of cohorts on basis of the design or analysis** | **Assessment of outcome** | **Follow-up long enough for outcomes to occur** | **Adequacy of follow-up of cohorts** |  |
| Aeschlimann et al, 2014 (20) | 1 | 0 | 1 | 0 | 0 | 1 | 0 | 0 | 3 |
| Alexeeva et al, 2017 (22) | 0 | 0 | 1 | 1 | 0 | 1 | 1 | 1 | 5 |
| Alexeeva et al, 2019 (23) | 0 | 0 | 1 | 1 | 0 | 1 | 1 | 1 | 5 |
| Armaroli et al, 2020 (21) | 1 | 1 | 1 | 0 | 0 | 1 | 1 | 1 | 6 |
| Bader-Meunier et al, 2019 (24) | 1 | 0 | 1 | 1 | 0 | 1 | 1 | 1 | 6 |
| Becker et al, 2017 (25) | 1 | 1 | 1 | 0 | 0 | 1 | 0 | 0 | 4 |
| Beukelman et al, 2016 (26) | 1 | 1 | 1 | 0 | 0 | 1 | 0 | 0 | 4 |
| Brunelli et al, 2020 (27) | 1 | 0 | 1 | 1 | 1 | 1 | 1 | 1 | 7 |
| Brunner et al, 2020 (28) | 1 | 1 | 1 | 0 | 0 | 1 | 1 | 1 | 6 |
| Cabrera et al, 2019 (29) | 1 | 1 | 1 | 0 | 0 | 1 | 0 | 0 | 4 |
| Choi et al, 2018 (30) | 1 | 0 | 1 | 0 | 0 | 1 | 1 | 1 | 5 |
| Constantine et al, 2016 (15) | 1 | 0 | 1 | 0 | 0 | 1 | 1 | 1 | 5 |
| Davies et al, 2015 (31) | 1 | 1 | 1 | 0 | 2 | 1 | 1 | 1 | 8 |
| Dumaine et al, 2020 (32) | 1 | 1 | 1 | 0 | 0 | 1 | 0 | 0 | 4 |
| Favalli et al, 2017 (33) | 1 | 1 | 1 | 0 | 0 | 1 | 1 | 1 | 6 |
| Feger et al, 2019 (34) | 1 | 0 | 1 | 0 | 0 | 1 | 1 | 1 | 5 |
| Foeldvari et al, 2019 (16) | 1 | 0 | 1 | 0 | 0 | 1 | 1 | 1 | 5 |
| Geikowski et al, 2014 (36) | 1 | 0 | 1 | 0 | 0 | 1 | 1 | 1 | 5 |
| Gerloni et al, 2008 (37) | 1 | 1 | 1 | 0 | 0 | 1 | 1 | 1 | 6 |
| Giannini et al, 2009 (38) | 1 | 1 | 1 | 0 | 0 | 1 | 1 | 1 | 6 |
| Goettel et al, 2021 (40) | 1 | 1 | 1 | 0 | 0 | 1 | 1 | 1 | 6 |
| Haapasaari et al, 2002 (41) | 1 | 0 | 1 | 0 | 0 | 1 | 1 | 1 | 5 |
| Halbig et al, 2009 (42) | 1 | 0 | 1 | 0 | 0 | 1 | 1 | 1 | 5 |
| Horneff et al, 2004 (43) | 1 | 1 | 1 | 0 | 0 | 1 | 1 | 1 | 6 |
| Horneff et al, 2009 (44) | 1 | 1 | 1 | 0 | 0 | 1 | 1 | 1 | 6 |
| Horneff et al, 2016 (45) | 1 | 1 | 1 | 0 | 2 | 1 | 1 | 1 | 8 |
| Horneff et al, 2017 (46) | 1 | 0 | 1 | 0 | 0 | 1 | 1 | 1 | 5 |
| Kearsley-Fleet et al, 2016 (49) | 1 | 0 | 1 | 0 | 2 | 1 | 1 | 1 | 7 |
| Kearsley-Fleet et al, 2016 (85) | 1 | 0 | 1 | 0 | 0 | 1 | 1 | 1 | 5 |
| Kimura et al, 2005 (50) | 1 | 0 | 1 | 0 | 0 | 1 | 1 | 1 | 5 |
| Kingsbury et al, 2014 (S18) | 1 | 0 | 1 | 0 | 0 | 1 | 1 | 1 | 5 |
| Klein et al, 2019 (51) | 1 | 1 | 1 | 0 | 2 | 1 | 1 | 1 | 8 |
| Klein et al, 2019 (52) | 1 | 1 | 1 | 0 | 2 | 1 | 1 | 1 | 8 |
| Klein et al, 2020 (53) | 1 | 1 | 1 | 0 | 0 | 1 | 1 | 1 | 6 |
| Klotsche et al, 2014 (54) | 1 | 0 | 1 | 0 | 0 | 1 | 1 | 1 | 5 |
| Klotsche et al, 2016 (55) | 1 | 1 | 1 | 0 | 0 | 1 | 1 | 1 | 6 |
| McErlane et al, 2013 (58) | 1 | 1 | 1 | 0 | 0 | 1 | 1 | 1 | 6 |
| Minden et al, 2012 (59) | 1 | 0 | 1 | 0 | 0 | 1 | 1 | 1 | 5 |
| Nielsen et al, 2008 (60) | 1 | 0 | 1 | 0 | 0 | 1 | 1 | 1 | 5 |
| Otten et al, 2011 (62) | 1 | 0 | 1 | 0 | 0 | 1 | 1 | 1 | 5 |
| Otten et al, 2013 (61) | 1 | 0 | 1 | 1 | 0 | 1 | 1 | 1 | 6 |
| Pastore et al, 2018 (63) | 1 | 0 | 1 | 0 | 0 | 1 | 0 | 1 | 4 |
| Prince et al, 2009 (65) | 1 | 0 | 1 | 0 | 0 | 1 | 1 | 1 | 5 |
| Prince et al, 2010 (64) | 1 | 0 | 1 | 0 | 0 | 1 | 1 | 1 | 5 |
| Quartier et al, 2003 (66) | 1 | 0 | 1 | 0 | 0 | 1 | 1 | 1 | 5 |
| Romano et al, 2014 (67) | 1 | 1 | 1 | 0 | 2 | 1 | 1 | 1 | 8 |
| Russo et al, 2009 (69) | 1 | 0 | 1 | 1 | 0 | 1 | 1 | 1 | 6 |
| Ruperto et al, 2021 (19) | 1 | 0 | 1 | 0 | 0 | 1 | 1 | 1 | 5 |
| Schmeling et al, 2014 (70) | 1 | 0 | 1 | 0 | 0 | 1 | 0 | 1 | 4 |
| Sevcic et al, 2011 (71) | 1 | 0 | 1 | 0 | 0 | 1 | 1 | 1 | 5 |
| Solari et al, 2013 (72) | 1 | 0 | 1 | 0 | 0 | 1 | 1 | 1 | 5 |
| Southwood et al, 2011 (73) | 1 | 0 | 1 | 0 | 0 | 1 | 1 | 1 | 5 |
| Su et al, 2017 (74) | 1 | 0 | 1 | 0 | 0 | 1 | 1 | 1 | 5 |
| Takei et al, 2021 (75) | 1 | 0 | 1 | 0 | 0 | 1 | 1 | 1 | 5 |
| Tambralli et al, 2013 (76) | 1 | 0 | 1 | 0 | 0 | 1 | 1 | 1 | 5 |
| Tarkiainen et al, 2015 (77) | 1 | 1 | 1 | 0 | 0 | 1 | 1 | 0 | 5 |
| Trachana et al, 2013 (78) | 1 | 0 | 1 | 0 | 0 | 1 | 1 | 1 | 5 |
| Tynjälä et al, 2009 (79) | 1 | 1 | 1 | 0 | 2 | 1 | 1 | 1 | 8 |
| Verazza et al, 2016 (80) | 1 | 0 | 1 | 0 | 0 | 1 | 1 | 1 | 5 |
| Windschall et al, 2015 (82) | 1 | 0 | 1 | 0 | 0 | 1 | 1 | 0 | 4 |
| Windschall et al, 2016 (83) | 1 | 0 | 1 | 0 | 0 | 1 | 1 | 0 | 4 |
| Zuber et al, 2011 (84) | 1 | 0 | 1 | 0 | 0 | 1 | 1 | 1 | 5 |

Interpretation of scores: 0–3 = poor, 4–5 = fair, ≥6 = good.

**Supplementary Table 11.** Efficacy and safety outcomes in observational studies of adalimumab

| **Registry, author, year** | **JIA category** | **Treatment and sample size** | **Time point (months)** | **JIA-ACR 30/50/70/90/100** | **JADAS, mean (standard deviation)** | **Remission,**  **n (%)** | **SAE^a^, n** | **SAE/ 100PY** |
| --- | --- | --- | --- | --- | --- | --- | --- | --- |
| ABIRISK/Pharmachild | | | | | | | | |
| Finetti, 2018 (35) | Mixed JIA | ADA: 80 | 0 | NR | JADAS10 Median (IQR) 12.6 (7 to 17) | NR | NR | NR |
|  |  | ADA: 80 | 3 | NR/NR/62.5/NR/NR | Median (IQR) 2.7 (1 to 5.9) | NR | NR | NR |
|  |  | ADA: 80 | 6 | NR/NR/67.2/NR/NR | Median (IQR) 1.5 (0 to 5) | NR | NR | NR |
|  |  | ADA: 80 | 12 | NR/NR/71.2/NR/NR | Median (IQR) 0.5 (0 to 5) | NR | NR | NR |
| BIKER | | | | | | | | |
| Becker, 2017 (25) | Mixed JIA | ADA: 60 | FU | NR | NR | NR | Number of serious infections 1 | 1.35 RR vs MTX *P* = 0.0514 |
|  |  | ADA+MTX: 117 | FU | NR | NR | NR | Number of serious infections  1 | 0.76 RR vs MTX *P* = 0.1558 |
|  |  | ADA±MTX: 177 | FU | NR | NR | NR | 2 serious infections | 0.97 RR vs MTX *P* = 0.0312 |
| Horneff, 2016 (45) | PA | ADA: 236 | 0 | NR | JADAS10  Mean (SD) 12.1 (7.6) *P* = 0.003 vs ETN OR (95% CI); *P* value ETN vs ADA ‑0.41 (‑2.30 to 1.48); 0.67 | NR | NR | NR |
|  | PA | ADA: 236 | 24 | NR | Change from baseline (95% CI) ‑7.3 (5.8 to 8.6) | 71 (27.9) | 26 (11%) | 11.0  RR 2.06 (1.35 to 3.16) ETN vs ADA |
| Horneff, 2019 (CA) (47) | Mixed JIA | ADA: 951 | 0 | NR | JADAS10 Median (IQR) 9.8 (4.7 to 15.5) | NR | NR | NR |
|  |  |  | LFU | NR | JADAS10 Median (IQR) 3.9 (0.9 to 9.7) | 238 (25) | 66 events | 4.3 |
|  |  | MTX: 1517 | LFU | NR | NR | NR | 52 events | 1.4 |
| Klein, 2019 (51) | Mixed JIA | ADA: 228 | 0 | NR | JADAS10  Mean (SD) 9.8 (6.8) | NR | NR | NR |
|  |  | ADA+MTX: 356 | 0 | NR | Mean (SD)  11.4 (7.3) | NR | NR | NR |
|  |  | ADA: 129 | 12 | 62/58.1/44.2/29.5/NR | n (%) patients with JADAS10 ≤3.8 61 (48.0) *P* < 0.001 vs baseline n (%) patients with JADAS10 ≤1 29 (22.5) | 29 (22.5) | NR | NR |
|  |  | ADA+MTX: 180 | 12 | 68.3/63.9/46.1/27.2/NR | n (%) patients with JADAS10 ≤3.8 94 (52.2) *P* < 0.001 vs baseline n (%) patients with JADAS10 ≤1 51 (28.3) | 51 (28.3) | NR | NR |
|  |  | ADA: 69 | 24 | 69.6/66.7/56.5/34.8/NR | n (%) patients with JADAS10 ≤3.8 42 (60.9) n (%) patients with JADAS10 ≤1 22 (31.9) | 22 (31.9) | NR | NR |
|  |  | ADA+MTX: 98 | 24 | 63.3/55.1/41.8/32.7/NR | n (%) patients with JADAS10 ≤3.8 55 (56.1) n (%) patients with JADAS10 ≤1 38 (38.8) | 38 (38.8) | NR | NR |
|  |  | ADA: 228 | LFU | 75.4/71.9/64/48.7/NR | n (%) JADAS10  6.2 (6.6) *P* < 0.001 vs baseline | 104 (45.6) | 27 events | 5.9 |
|  |  | ADA+MTX: 356 | LFU | 77.5/71.9/60.7/44.7/NR | n (%) JADAS10  6.1 (6.5)  *P* < 0.001 vs baseline  Improvement from baseline vs ADA mono: *P* = 0.02 | 169 (47.5)  HR (95% CI) 1.10 (0.85 to 1.44) | 30 events | 4.8 |
|  |  | ADA±MTX: 584 | LFU | NR | NR | NR | 57 events | 5.3 |
| Klein, 2020 (53) | PA | ADA: 828 | LFU | NR | NR | NR | 27 events | 1.86 |
| Schmeling, 2014 (70) | Mixed JIA | All ADA: 289 | 0 | NR | JADAS10 Mean (median) 10.1 (10.8) | NR | NR | NR |
|  |  | ADA (biologic-naïve): 130 | 0 | NR | JADAS10  Mean (median) 12.8 (12.9) | NR | NR | NR |
|  |  | ADA (biologic switcher): 159 | 0 | Prior to ADA initiation  60/51/33.1/15.9/NR  (n = 145) | JADAS10  Mean (median) 9.2 (8.5) *P* < 0.001 vs biologic-naïve group | NR | NR | NR |
|  |  | ADA (biologic-naïve): 130 | 6 | 63.4/61/48.8/34.2/NR | NR | NR | NR | NR |
|  |  | ADA (biologic switcher): 159 | 6 | 47.6/38.1/21.9/15.2/NR | NR | NR | NR | NR |
|  |  | ADA (biologic-naïve): 130 | LFU | NR | NR | NR | 6 SAEs | 3.2 |
|  |  | ADA (biologic switcher): 159 | LFU | NR | NR | NR | 5 SAEs | 2.0 |
|  |  | All ADA: 289 | LFU | NR | NR | NR | 11 SAEs | 2.5 |
| BIKER/JuMBO | | | | | | | | |
| Klotsche, 2016 (55) | Mixed JIA | ADA: 46 | LFU | NR | NR | NR | 23 events | 4.67  RR (95% CI); *P* value ADA vs MTX 2.17 (1.25 to 3.77); *P* = 0.006 ADA vs ETN 1.00 (0.60 to 1.65); *P* = 0.988 |
|  |  | MTX: 1055 | LFU | NR | NR | NR | 75 events | 2.58 |
| JIRcohorte | | | | | | | | |
| Cabrera, 2019 (29) | Mixed JIA | ADA: NR | NR | NR | NR | NR | SAEs: 5 Very SAEs: NR Hospitalization: 3 | SAEs: 0.8 Very SAEs: NR Hospitalization: 0.5 |
| Dumaine, 2020 (32) | Mixed JIA | ADA: 203 | NR | NR | NR | NR | Severe  1 (5.2)  Very severe  0  Hospitalization 1 (5.3) | Severe  0.2  Very severe  0  Hospitalization  0.2 |
| STRIVE | | | | | | | | |
| Brunner, 2020 (28) | Mixed JIA | ADA: 160 | FU | NR | NR | NR | 39 events  SAE Infection: 8   SAE at least possibly drug related: 9 | 7.5  Infection: 1.5   SAE at least possibly drug related:1.7 |
|  |  | ADA+MTX: 377 | FU | NR | NR | NR | 95 events   SAE Infection: 30  SAE at least possibly drug related: 23 | 7.1  Infection 2.2  SAE at least possibly drug related: 1.7 |
|  |  | ADA+/-MTX: 537 | FU | NR | NR | NR | 134 events   SAE Infection: 38  SAE at least possibly drug related: 32 | 7.2  SAE infection 2.0  SAE at least possibly drug related 1.7 |
|  |  | ADA (prior biologic DMARD): 57 | FU | NR | NR | NR | 15 events  SAE Infection: 5  SAE at least possibly drug related: 3 | 8.2  SAE infection 2.7  SAE at least possibly drug related  1.6 |
|  |  | ADA+MTX (prior biologic DMARD): 105 | FU | NR | NR | NR | 21 events  SAE Infection: 4  SAE at least possibly drug related: 3 | 5.9  SAE infection 1.1  SAE at least possibly drug related  0.8 |
|  |  | ADA+/-MTX (prior biologic DMARD): 160 | FU | NR | NR | NR | 36 events   SAE Infection: 9  SAE at least possibly drug related: 6 | 6.7  SAE infection 1.7  SAE at least possibly drug related  1.1 |

| Registry NR | | | | | | | | |
| --- | --- | --- | --- | --- | --- | --- | --- | --- |
| Beukelman, 2016 (26) | Mixed JIA | ADA: NR | FU | NR | NR | NR | Number of events (infections) requiring hospitalization: 12 | Serious infection rate  2.90   Unadjusted HR vs ETN (95% CI) 1.99 (1.03 to 3.87)  Adjusted HR vs ETN (95% CI) 2.39 (1.21 to 4.72)  Adjusted HR for time period vs ETN (95% CI) 1.39 (0.62 to 3.19) |
| Goettel, 2021 (40) | Mixed JIA | ETN: 79  ADA: 49 | 4–6 | NR/NR/NR/NR/NR  NR/NR/NR/NR/NR | NR  NR | NR  NR | NR  NR | NR  NR |
| Takei, 2021 (75) | Mixed JIA | ADA: 356 | 6  LFU | NR/NR/NR/NR/NR  NR/NR/NR/NR/NR | NR  NR | 112 (74.7)  145 (71.4) | NR  239 | NR  NR |
| Tarkiainen, 2015 (77) | Mixed JIA | ADA: 94 | NR | NR | NR | NR | Serious infection AE n(%) 3 (3.2) | 10.1 Serious infection AE 2.1 |
| Verstegen, 2020 (81) | JIA (NR) | ETN: 296  ADA: 406 | NR  NR | 72/63/50/34/30  50/46/31/20/19 | NR  NR | NR  NR | NR  NR | NR  NR |

**Supplementary Table 12.** Efficacy and safety outcomes in observational studies of etanercept

| **Registry, author, year** | **JIA category** | **Treatment and sample size** | **Time point (months)** | **JIA-ACR 30/50/70/90/100** | **JADAS, mean (standard deviation)** | **Remission,**  **n (%)** | **SAE^a^, n** | **SAE/ 100PY** |
| --- | --- | --- | --- | --- | --- | --- | --- | --- |
| ABC | | | | | | | | |
| Otten, 2013 (61) | Mixed JIA | ETN: 307 | 12 | NR | NR | NR | NR | 0.01* |
|  |  |  | LFU | NR | NR | NR | NR | 0.01* |
| Otten, 2011 (62) | Mixed JIA | ETN: 85 | 15 | NR | NR | 81 (95.3) | NR | NR |
|  | Mixed JIA | ETN: 262 | LFU | NR | NR | NR | 31 events | 0.05* |
| Prince, 2010 (64) | Mixed JIA | ETN: 53 | 27 | NR | NR | NR | 1 (1.9%) | 0.01* |
| Prince, 2009 (65) | Mixed JIA | ETN: 146 | 0 | NR | NR | NR | NR | NR |
|  |  | ETN: 142 | 3 | 78.9/66.9/50.7/NR/NR | NR | 0 | NR | NR |
|  |  | ETN: 97 | 15 | 92.8/90.7/76.3/NR/NR | NR | 35 (36.1) | NR | NR |
|  |  | ETN: 57 | 27 | 87.7/80.7/70.2/NR/NR | NR | 27 (47.4) | NR | NR |
|  |  | ETN: 146 | 75 | NR | NR | 53 (36) | 9 (6.2%) | 0.029* |
|  | sJIA | ETN: 37 | 3 | 59.5/43.2/27.0/NR/NR | NR | NR | NR | NR |
|  |  | ETN: 39 | 75 | NR | NR | 14 (38) | NR | NR |
|  | RF– PA | ETN: 53 | 3 | 90.6/79.2/62.3/NR/NR | NR | NR | NR | NR |
|  | RF– PA | ETN: 36 | 15 | 91.7/91.7/77.8/NR/NR | NR | 38.9 | NR | NR |
|  | RF– PA | ETN: 55 | 75 | NR | NR | 20 (38) | NR | NR |
| ABIRISK/Pharmachild | | | | | | | | |
| Finetti, 2018 (35) | Mixed JIA | ETN: 36 | 0 | NR | JADAS10 Median (IQR) 15.6 (10 to 18.5) | NR | NR | NR |
|  |  |  | 3 | NR/NR/63/NR/NR | JADAS10 Median (IQR) 2.2 (0 to 7.7) | NR | NR | NR |
|  |  |  | 6 | NR/NR/62.1/NR/NR | JADAS10 Median (IQR) 1 (0 to 8) | NR | NR | NR |
|  |  |  | 12 | NR/NR/76/NR/NR | JADAS10 Median (IQR) 0.5 (0 to 4.2) | NR | NR | NR |
| ANTARES | | | | | | | | |
| Nielsen, 2008 (60) | Mixed JIA | ETN: 40 | 0 | NR | NR | NR | NR | NR |
|  |  |  | 3 | 72.2/NR/NR/NR/NR  n = 36 | NR | NR | NR | NR |
|  |  |  | 6 | 66.7/NR/NR/NR/NR  n = 36 | NR | NR | NR | NR |
|  |  |  | 12 | 77.0/72/50/NR/NR  n = 31 | NR | NR | NR | NR |
| BIKER | | | | | | | | |
| Armaroli, 2020 (21) | Mixed JIA | ETN: 2725 | 3  12  24  60  84  108  216 | 74/64/45/24/19  81/75/61/24/27  82/76/64/57/25  84/80/68/53/20  82/79/69/57/19  82/79/71/54/19  NR/NR/NR/NR/NR | JADAS10: 5.6 (5.7) *P* < 0.0001 vs BL  JADAS10: 4.1 (5.3)  *P* < 0.0001 vs BL  NR  NR  NR  NR  NR | 315 (17.2)  591 (35.0)  449 (37.8)  175 (43.8)  76 (40.4)  31 (43.1)  NR | NR  NR  NR  NR  NR  NR  226 | NR  NR  NR  NR  NR  NR  3.8 |
| Becker, 2017 (25) | Mixed JIA | ETN: 526 courses | FU | NR | NR | NR | Events of serious infections: 5 | 0.52  RR vs MTX *P* = 0.0611 |
|  |  | ETN+MTX: 1194 courses | FU | NR | NR | NR | Events of serious infections: 16 | 0.98 RR vs MTX *P* = 0.0004 |
|  |  | ETN±MTX: 1720 courses | FU | NR | NR | NR | Events of serious infections: 21 | 0.81  RR vs MTX *P* = 0.0011 |
| Geikowski, 2014 (36) | Mixed JIA | ETN: 863 | 0 | NR | NR | NR | NR | NR |
|  |  |  | 6 | 81.9/NR/55.2/31.3/NR | NR | NR | NR | NR |
|  |  | ETN: 707 | 0 | 100/NR/NR/NR/NR | NR | NR | NR | NR |
|  |  | ETN: 156 | 0 | 0/NR/NR/NR/NR | NR | NR | NR | NR |
|  |  | ETN: 476 | 0 | NR/NR/100/NR/NR | NR | NR | NR | NR |
|  |  | ETN: 387 | 0 | NR/NR/0/NR/NR | NR | NR | NR | NR |
|  |  | ETN: 270 | 0 | NR/NR/NR/100/NR | NR | NR | NR | NR |
|  |  | ETN: 593 | 0 | NR/NR/NR/0/NR | NR | NR | NR | NR |
| Halbig, 2009 (42) | Mixed JIA | ETN: 437 | 0 | NR | NR | NR | NR | NR |
|  |  | ETN: 114 | 24 | 96.5/93.8/90.3/NR/NR | NR | NR | NR | NR |
| Horneff, 2004 (43) | Mixed JIA | ETN: 322 | 3 | 76/61/39/NR/NR | NR | NR | NR | NR |
|  |  |  | 6 | 82/70/50/NR/NR | NR | NR | NR | NR |
|  |  |  | 12 | 80/71/54/NR/NR | NR | NR | NR | NR |
|  |  |  | 30 | NR | NR | 72 (26) | 12 (3.7%) | NR |
|  | sJIA | ETN: 66 | 3 | 63/39/24/NR/NR | NR | NR | NR | NR |
|  |  | ETN: 66 | 30 | NR | NR | 8 (13) | NR | NR |
|  | RF– PA | ETN: 94 | 30 | NR | NR | 14 (17) | NR | NR |
|  | RF+ PA | ETN: 39 | 30 | NR | NR | 11 (30) | NR | NR |
|  | ExOA | ETN: 54 | 30 | NR | NR | 14 (28) | NR | NR |
|  | nsJIA | ETN: 256 | 3 | 79/NR/NR/NR/NR | NR | NR | NR | NR |
|  |  | ETN: 256 | 12 | 90/82/64/NR/NR | NR | NR | NR | NR |
|  |  | ETN: 256 | 30 | NR | NR | 31% *P* < 0.02 vs sJIA | NR | NR |
|  | Mixed JIA | ETN+MTX: 235 | 30 | NR | NR | 29% | NR | NR |
|  |  | ETN mono: 87 | 30 | NR | NR | 14% *P* = 0.07 vs ETN+MTX | NR | NR |
| Horneff, 2009 (44) | Mixed JIA | ETN: 100 | 0 | NR | NR | NR | NR | NR |
|  |  |  | 12 | 70/63/45/NR/NR  n = 67 | NR | NR | 4 (4%) n = 100 | 0.04 |
|  |  | ETN+MTX: 540 | 0 | NR | NR | NR | NR | NR |
|  |  | ETN+MTX: 376 | 12 | 81/74/62/NR/NR  n = 419 | NR | NR | 48 (9.5%) n = 504 | NR |
| Horneff, 2016 (45) | Mixed JIA | ETN: 419 | 0 | NR | JADAS10 138 (7.1) | NR | NR | NR |
|  | Mixed JIA | ETN: 419 | 24 | NR | Change from baseline (95% CI): ‑8.6 (-7.6 to -9.5) | 131 (34.8) | 119 (28%) | 22.07 |
| Horneff, 2017 (46) | sJIA | ETN: 143 | 0 | NR | JADAS10 Median (IQR)  20.8 (14.0 to 28.4) | NR | NR | NR |
|  |  |  | 3 | NR/NR/32/16/NR | Median (IQR) 6.9 (2.5 to 14.3) | 10 (11)  n = 93 | NR | NR |
|  |  |  | 6 | NR/NR/35/19/NR | Median (IQR) 6.2 (1.1 to 14.7) | 21 (20)  n = 105 | NR | NR |
|  |  |  | 12 | NR/NR/36/22/NR | Median (IQR) 3.8 (0.7 to 15.7) | 22 (20)  n = 109 | NR | NR |
|  |  |  | 18 | NR/NR/33/18/NR | Median (IQR) 3.8 (0.7 to 15.7) | 16 (15)  n = 110 | NR | NR |
|  |  |  | 24 | NR/NR/34/19/NR | Median (IQR)  3.3 (0.7 to 9.4) | 18 (17)  n = 109 | NR | NR |
|  |  |  | LFU | NR | Median (IQR) 9.1 (2.1; 19.1) | NR | 18 (13%) n = 143 | 0.05* |
| Klein, 2020 (86) | sJIA | ETN: 151 | LFU | NR | NR | NR | 9 (6.0%) 14 events | 3.53 RR (95% CI): 0.51 (0.21 to 1.22); *P* = 0.129 |
|  | sJIA | ETN: NR | LFU | NR | NR | NR | 2 (1.3%) 2 events | 1.29 RR (95% CI): 0.26 (0.05 to 1.45); *P* = 0.124 |
|  | sJIA | ETN+ss: NR | LFU | NR | NR | NR | 7 (4.6%) 12 events | 4.96 RR (95% CI): 0.61 (0.19 to 1.96); *P* = 0.403 |
| Klein, 2019 (CA) (52) | MIXED JIA | ETN: 2645 | 0 | NR | JADAS10 Median (IQR) 15.0 (10 to 20.4) | NR | NR | NR |
|  |  |  | LFU | 68/62/50/34/NR | JADAS10 Median (IQR) 3.5 (0.7 to 10.1) | 1058 (40) | 221 events | 3.8 |
| Klein, 2020 (53) | PA | ETN: 2338 | LFU | NR | NR | NR | 109 events | 2.02  RR (95% CI): 0.78 (0.56 to 1.08) |
| Windschall, 2016 (83) | PA | ETN: 74 | 6 | NR | NR | 26 (35) | NR | NR |
|  |  |  | 12 | NR | NR | 33 (44) | NR | NR |
|  |  |  | 24 | NR/64/54/41/NR | NR | 37 (50) | 3 SAEs | 1.9 |
| Windschall, 2015 (82) | ERA | ETN: 238 | 24 | NR | NR | NR | 17 events | 4.19 |
|  | PsA | ETN: 127 | 24 | NR | NR | NR | 11 events | 5.19 |
|  | ExOA | ETN: 315 | 24 | NR | JADAS10 3.0 (3.5) n=102 *lowest mean JADAS at 24 months* | NR | 31 events | 4.54 |
|  | RF– PA | ETN: 534 | 24 | NR | NR | NR | 33 events | 2.91 |
|  | RF+ PA | ETN: 160 | 24 | NR | NR | NR | 16 events | 4.3 |
| BIKER/JuMBO | | | | | | | | |
| Klotsche, 2016 (55) | Mixed JIA | ETN: 1162 | LFU | NR | NR | NR | 199 events | 4.46  RR (95% CI);  *P* value ETN vs MTX 2.18 (1.56 to 3.06); *P* < 0.001 ADA vs ETN 1.00 (0.60 to 1.65); *P* = 0.988 |
|  |  | ETN: NR | LFU | NR | NR | NR | 125 events | 5.28 |
|  |  | ETN: NR | LFU | NR | NR | NR | 189 events | 4.52 |
| Klotsche, 2019 (CA) (56) | Mixed JIA | ETN: 1765 | NR | NR | NR | NR | 218 (7.3%) 320 events | 4.76 |
|  |  | ETN: 518 | NR | NR | NR | NR | 116 (22.4%) 189 events | 4.17 |
| Klotsche, 2020 (57) | Mixed JIA (NR) | ETN: 1724 | NR | NR/NR/NR/NR/NR | NR | NR | NR | NR |
| BSPAR-ETN | | | | | | | | |
| Davies, 2015 (31) | Mixed JIA | ETN: 852 | NR | NR | NR | NR | Based on first event: 46 events | Crude incidence rates of serious infection (95% CI) 2.2 (1.6 to 3.0) Unadj HR (95% CI) for first serious infection vs MTX mono: 1.18 (0.59 to 2.35) vs ETN mono: NR Fully adj HR (95% CI) for first SERIOUS INFECTION vs MTX mono: 1.36 (0.60 to 3.07) vs ETN mono: NR |
|  |  | ETN: 399 | NR | NR | NR | NR | Based on first event: 22 events | Crude incidence rates of MSI (95% CI) 1.8 (1.1 to 2.8) Unadj HR (95% CI) for first MSI vs MTX mono: 1.06 (0.49 to 2.27) vs ETN mono: Reference Fully adj HR (95% CI) for first MSI vs MTX mono: 1.29 (0.48 to 3.50) vs ETN mono: Reference |
|  |  | ETN+MTX: 453 | NR | NR | NR | NR | Based on first event: 24 events | Crude incidence rates of MSI (95% CI)  2.8 (1.8 to 4.2) Unadj HR (95% CI) for first MSI vs MTX mono: 1.21 (0.57 to 2.58) vs ETN mono: 1.23 (0.66 to 2.29) Fully adj HR (95% CI) for first MSI vs MTX mono: 1.30 (0.51 to 3.30) vs ETN mono: 1.29 (0.63 to 2.62) |
| Kearsley-Fleet, 2016 (49) | Mixed JIA | ETN: 496 | 0 | NR | JADAS71 Median (IQR) Table 1 16.9 (12.0 to 24.5)  n = 218 Table 3 16.6 (12.0 to 24.1) n = 496 | NR | NR | NR |
|  |  | ETN: 496 | 12 | 74/69/56/38/NR | JADAS71 median (IQR) Table 1 3.8 (0.8 to 9.0)  n = 237 Table 2  3.7 (0.6 to 9.3)  n = 496 *P* < 0.001 from baseline | NR | NR | NR |
|  | sJIA | ETN: 73 | 0 | NR | JADAS71 median (IQR) 22.3 (15.5 to 33.8) | NR | NR | NR |
|  |  |  | 12 | 69/64/48/27/NR | JADAS71  median (IQR) 5.3 (1.1 to 11.9) *P* < 0.001 vs baseline | NR | NR | NR |
|  | ExOA | ETN: 81 | 0 | NR | JADAS71 Median (IQR) 14.6 (9.0 to 19.2) | NR | NR | NR |
|  |  |  | 12 | 76/70/58/38/NR | JADAS71  Median (IQR) 3.7 (1.0 to 8.0)  *P* < 0.001 vs baseline | NR | NR | NR |
|  | RF– PA | ETN: 181 | 0 | NR | JADAS71 Median (IQR) 17.0 (11.7 to 24.6) | NR | NR | NR |
|  |  |  | 12 | 76/71/60/39/NR | JADAS71 Median (IQR) 3.2 (0.7 to 9.3) *P* < 0.001 vs baseline | NR | NR | NR |
|  | RF+ PA | ETN: 43 | 0 | NR | JADAS71 Median (IQR) 17.3 (13.8 to 22.9) | NR | NR | NR |
|  |  |  | 12 | 62/56/43/37/NR | JADAS71 Median (IQR) 6.3 (0.7 to 13.5)  *P* < 0.001 | NR | NR | NR |
|  | PsA | ETN: 36 | 0 | NR | JADAS71  Median (IQR) 17.5 (12.4 to 24.8) | NR | NR | NR |
|  |  |  | 12 | 60/58/50/39/NR | JADAS71  Median (IQR) 6.1 (1.0 to 14.3) *P* < 0.05 vs baseline | NR | NR | NR |
|  | ERA | ETN: 35 | 0 | NR | JADAS71  Median (IQR) 17.2 (12.4 to 22.0) | NR | NR | NR |
|  |  |  | 12 | 89/84/68/45/NR | JADAS71 Median (IQR) 3.0 (0.5 to 5.9) *P* < 0.001 vs baseline | NR | NR | NR |
|  | Undiff | ETN: 35 | 0 | NR | JADAS71  Median (IQR) 17.1 (11.6 to 28.5) | NR | NR | NR |
|  |  |  | 12 | 83/79/60/45/NR | JADAS71 Median (IQR) 1.7 (0.04 to 6.7)  *P* < 0.001 vs baseline | NR | NR | NR |
| BSPAR and BNDR | | | | | | | | |
| Southwood, 2011 (73) | Mixed JIA | ETN: 483 | 60 | NR | NR | NR | NR | NR |
| EtICA | | | | | | | | |
| Verazza, 2016 (80) | Mixed JIA | ETN: 1038 | 60 | NR | NR | NR | NR | NR |
|  |  | ETN (cross-sectional cohort): 422 | NR | NR | Inactive disease,  Wallace criteria:  164 (41.8);  JADAS10:  196 (46.4);  cJADAS10:  205 (48.6) | NR | NR | NR |
|  |  | ETN (retrospective cohort): 616 | NR | NR | NR | 344 (55.8) | NR | NR |
| JIRcohorte | | | | | | | | |
| Cabrera, 2019 (29) | Mixed JIA | ETN: NR | NR | NR | NR | NR | Events  SAEs: 11 Very SAEs: 2 Hospitalization: 11 | Incidence rate  SAEs 0.7  Very SAEs 0.1  Hospitalization  0.7 |
| Dumaine, 2020 (32) | Mixed JIA | ETN: 506 | NR | NR | NR | NR | n (%)/100PY Severe  3 (4.1%)/0.2 Very severe  0 Hospitaliz-ation 10 (13.7%)/ 0.7  25% of severe or very severe IAEs for whole cohort 42% of hospitaliz-ations for whole study cohort | NR |
| JuMBO | | | | | | | | |
| Minden, 2012 (59) | Mixed JIA | ETN: 346 | LFU | NR | NR | NR | NR | 5.7 |
| NIRPR | | | | | | | | |
| Sevcic, 2011 (71) | Mixed JIA | ETN: 72 | 0 | NR | NR | NR | NR | NR |
|  |  | ETN: 71 | 3 | 88/80/40/10/NR | NR | NR | NR | NR |
|  |  | ETN: 61 | 12 | 76/66/56/37/NR | NR | NR | NR | NR |
|  |  | ETN: 72 | LFU | NR | NR | 2 (18) | NR | NR |
| Polish JIA Registry | | | | | | | | |
| Zuber, 2011 (84) | Mixed JIA | ETN: 188 | 0 | NR | NR | NR | NR | NR |
|  |  | ETN: 172 | 3 | 81.4/65.9/27.5/16.2/15.0  n = 167 | NR | NR | NR | NR |
|  |  | ETN: 158 | 6 | 86.3/78.4/35.9/16.3/14.4  n = 153; *P* < 0.0018 vs previous assessment | NR | NR | NR | NR |
|  |  | ETN: 146 | 12 | 90.5/86.5/53.9/22.7/19.1  n = 141; *P* < 0.0000 vs previous assessment | NR | NR | NR | NR |
|  |  | ETN: 97 | 24 | 94.7/88.4/62.1/34.7/26.3  n = 95; *P* < 0.0003 vs previous assessment | NR | NR | NR | NR |
|  |  | ETN: 58 | 36 | 89.3/82.1/51.8/21.4/17.9  n = 56; *P* < 0.0018 | NR | NR | NR | NR |
|  |  | ETN: 39 | 48 | 97.4/92.1/68.4/23.7/23.7  n = 38 | NR | NR | NR | NR |
|  |  | ETN: 188 | 72 | NR | NR | NR | 5 (3%) 6 events | 0.02 |
| Registry NR | | | | | | | | |
| Alexeeva, 2017 (22) | Mixed JIA | ETN: 197 | 12 | 90.9/89.9/85.3/68.5/NR | JADAS71 Median (IQR) 1.1 (0.3 to 3.9) | NR | NR | NR |
|  | POA | ETN: 84 | 12 | 90.5/90.5/88.1/77.4/NR | JADAS71  Median (IQR) 0.5 (0 to 1.1) | NR | NR | NR |
|  | RF– PA | ETN: 64 | 12 | 90.6/87.5/85.4/57.8/NR | JADAS71  Median (IQR) 1.45 (0.65 to 6.4) | NR | NR | NR |
| Alexeeva, 2019 (23) | Mixed JIA | ETN: 91 | 3 | 97.8/85.7/65.9/45.1/NR | NR | 29 (31.9); *P* < 0.001 vs other arms | NR | NR |
|  |  |  | 6 | 98.9/94.5/85.7/60.4/NR | NR | 48 (52.7); *P* < 0.001 vs other arms | NR | NR |
|  |  |  | 12 | 97.8/94.5/89.0/73.6/NR | NR | 50 (54.9); *P* = 0.029 vs other arms | NR | NR |
| Bader-Meunier, 2019 (24) | Mixed JIA | ETN: 126 | 0 | NR | Median (range) 1 (0 to 21) | Inactive disease, % (95% CI): 72 (61 to 81) | NR | NR |
|  |  | ETN: 104 | Visit 2 | NR | Median (range) 0.7 (0 to 16) | Inactive disease, % (95% CI): 75 (64 to 84) | 0 (0%) | NR |
| Beukelman, 2016 (26) | JIA (NR) | ETN: NR | FU | NR | NR | NR | Number of infections requiring hospitalization 37 | Serious infections/ 1.43 |
| Choi, 2018 (30) | Active PA JIA (NR) | ETN: 83 | NR | NR | NR | NR | Severe ADE 1 (0.9%) | NR |
| Gerloni, 2008 (37) | MIXED JIA | ETN: 127 treatments | NR | NR/NR/NR/NR/NR | NR | NR | 2 serious infections | NR |
| Giannini, 2009 (38) | Mixed JIA | ETN+MTX: 270 | 6 | NR | NR | NR | NR | NR |
|  |  | ETN: 95 | 6 | NR | NR | NR | NR | NR |
| Glazyrina, 2019 (CA) (39) | Mixed JIA | ETN: 51 | 12 | 3.9/9.8/21.6/62.7/NR | NR | 32 (62.7) | NR | NR |
| Goettel, 2021 (40) | Mixed JIA | ETN: 79  ADA: 49 | 4–6 | NR/NR/NR/NR/NR  NR/NR/NR/NR/NR | NR  NR | NR  NR | NR  NR | NR  NR |
| Kimura, 2005 (50) | sJIA | ETN: 82 | LFU | Modified response (not ACR)  8.5/13.4/32.9/NR/NR | NR | NR | 2 (2%) | NR |
| Pastore, 2018 (63) | Mixed JIA | ETN: 64 | LFU | NR | NR | NR | 8 events | 4.14 |
| Quartier, 2003 (66) | Mixed JIA | ETN: 61 | 3 | Improvement in core-set score from baseline  73/54/38/NR/NR | NR | NR | NR | NR |
|  |  |  | 6 | Improvement in core-set score from baseline  61/52/33/NR/NR | NR | NR | NR | NR |
|  |  |  | 9 | Improvement in core-set score from baseline  51/44/33/NR/NR | NR | NR | NR | NR |
|  |  |  | 12 | Improvement in core-set score from baseline  39/35/26/NR/NR | NR | NR | NR | NR |
| Russo, 2009 (69) | sJIA | ETN: 45 | 6 | NR | NR | 8 (18) | NR | NR |
| Solari, 2013 (72) | Mixed JIA | ETN: 173 | LFU | NR | NR | 70 (45) | Serious infections 3 (1.7%) | NR |
| Su, 2017 (74) | Mixed JIA | ETN: 58 | NR | NR | NR | 30 (52) | NR | NR |
|  |  | ETN: 30 | Last visit | NR/NR/86.7/NR/NR | NR | NR | NR | NR |
| Tarkiainen, 2015 (77) | Mixed JIA | ETN: 213 | NR | NR | NR | NR | Serious infection AE  21 (9.9%) | 11.4  Serious infection 4.2 |
| Trachana, 2013 (78) | Mixed JIA | ETN: 41 | 0 | NR | Median (IQR) 19.4 (12.1 to 27.1) | NR | NR | NR |
|  |  |  | 12 | 100/90.5/71.4/57.1/40.5 | JADAS71  1.45 *P* < 0.0001 vs baseline | Annual clinical remission of the ETN receivers n (%) 21 (50) Sustained clinical remission of the ETN receivers n (%) 21 (50) | NR | NR |
|  |  |  | 24 | 97.6/92.9/83.3/61.9/45.2 | NR | Annual clinical remission of the ETN receivers n (%) 24 (54.14) Sustained clinical remission of the ETN receivers n (%) 24 (54.14) | NR | NR |
| Verstegen, 2020 (81) | JIA (NR) | ETN: 296  ADA: 406 | NR  NR | 72/63/50/34/30  50/46/31/20/19 | NR  NR | NR  NR | NR  NR | NR  NR |

**Supplementary Table 13.** Efficacy and safety outcomes in observational studies of golimumab

| **Registry, author, year** | **JIA category** | **Treatment and sample size** | **Time point (months)** | | **JIA-ACR 30/50/70/90/100** | **JADAS, mean (standard deviation)** | **Remission,**  **n (%)** | **SAE^a^, n** | **SAE/ 100PY** |
| --- | --- | --- | --- | --- | --- | --- | --- | --- | --- |
| BIKER | | | | | | | | | |
| Klein, 2020 (53) | PA | GOL: 86 | | LFU | NR | NR | NR | 5 events | 5.32   RR (95% CI) 2.48 (1.02 to 6.04) |
| Horneff, 2020 (48) | Mixed JIA | GOL: 55  MTX: 47  GOL: 55  MTX: 47 | | 6  12 | 56/56/35/21/NR  NR/NR/NR/NR/NR  NR/NR/NR/NR/NR  NR/NR/NR/NR/NR | JADAS10: 6.4  NR  NR  NR | 10 (18)  NR  16 (29)  NR | NR  2  NR  1 | NR  4.2  NR  2.7 |
| JIRcohorte | | | | | | | | | |
| Cabrera, 2019 (29) | Mixed JIA | GOL: NR | | NR | NR | NR | NR | NR | NR |
| Registry NR | | | | | | | | | |
| Beukelman, 2016 (26) | Mixed JIA | GOL: NR | | FU | NR | NR | NR | Number of infections requiring hospitalization 0 | serious infection rate  0 |
| Ruperto, 2019 (68) | PA | GOL: 127 | | 7 | 84/80/70/47/NR | Change from baseline JADAS10 ‑14.20 JADAS27 -16.60 JADAS71 -20.32 | inactive disease 37 (29.1) | 6 (4.7%) | NR |

**Supplementary Table 14.** Efficacy and safety outcomes in observational studies of infliximab

| **Registry, author, year** | **JIA category** | **Treatment and sample size** | **Time point (months)** | **JIA-ACR 30/50/70/90/100** | **JADAS, mean (standard deviation)** | **Remission,**  **n (%)** | **SAE^a^, n** | **SAE/ 100PY** |
| --- | --- | --- | --- | --- | --- | --- | --- | --- |
| BIKER | | | | | | | | |
| Klein, 2020 (53) | PA | INF: 63 | LFU | NR | NR | NR | 8 events | 7.39   RR (95% CI) 3.51 (1.72 to 7.14) |
| JIRcohorte | | | | | | | | |
| Cabrera, 2019 (29) | Mixed JIA | INF: NR | NR | NR | NR | NR | Events  SAEs: 16 Very SAEs: 1 Hospitalization: 4 | SAEs 3.4  Very SAEs 0.2  Hospitalization  0.8 |
| Dumaine, 2020 (32) | Mixed JIA | INF: 138 | NR | NR | NR | NR | n(%)/100PY Severe  2 (6.9%)/0.4 Very severe 0  Hospitalization 1 (3.4%)/0.2  16% of severe or very severe IAEs for whole cohort 4% of hospitalizations for whole study cohort | NR |
| Registry NR | | | | | | | | |
| Beukelman, 2016 (26) | Mixed JIA | INF: NR | FU | NR | NR | NR | Number of infections requiring hospitalization 3 | Serious infection rate 1.32 |
| Gerloni, 2008 (37) | Mixed JIA | INF: 81 treatments | NR | NR | NR | NR | 1 serious infection | NR |
| Tambralli, 2013 (76) | Mixed JIA | INF: 58 | 12 | NR | NR | NR | 8 (13.8%)  9 events | 9.5 |
| Tarkiainen, 2015 (77) | Mixed JIA | INF: 214 | NR | NR | NR | NR | Serious infection AE 19 (8.9%) | 11.8 Serious infection  3.4 |

**Supplementary References**

1. Alexeeva E, Horneff G, Dvoryakovskaya T, Denisova R, Nikishina I, Zholobova E, et al. Early combination therapy with etanercept and methotrexate in JIA patients shortens the time to reach an inactive disease state and remission: results of a double-blind placebo-controlled trial. Pediatr Rheumatol Online J. 2021;19(1):5.

2. Brunner HI, Ruperto N, Tzaribachev N, Horneff G, Chasnyk VG, Panaviene V, et al. Subcutaneous golimumab for children with active polyarticular-course juvenile idiopathic arthritis: results of a multicentre, double-blind, randomised-withdrawal trial. Ann Rheum Dis. 2018;77(1):21–9.

3. Burgos-Vargas R, Tse SM, Horneff G, Pangan AL, Kalabic J, Goss S, et al. A randomized, double-blind, placebo-controlled multicenter study of adalimumab in pediatric patients with enthesitis-related arthritis. Arthritis Care Res (Hoboken). 2015;67(11):1503–12.

4. Hissink Muller PC, Brinkman DM, Schonenberg D, Koopman-Keemink Y, Brederije IC, Bekkering WP, et al. A comparison of three treatment strategies in recent onset non-systemic juvenile idiopathic arthritis: initial 3-months results of the BeSt for Kids-study. Pediatr Rheumatol Online J. 2017;15(1):11.

5. Horneff G, Foeldvari I, Minden K, Trauzeddel R, Kümmerle-Deschner JB, Tenbrock K, et al. Efficacy and safety of etanercept in patients with the enthesitis-related arthritis category of juvenile idiopathic arthritis: results from a phase III randomized, double-blind study. Arthritis Rheumatol. 2015;67(8):2240–9.

6. Lovell DJ, Giannini EH, Reiff A, Cawkwell GD, Silverman ED, Nocton JJ, et al. Etanercept in children with polyarticular juvenile rheumatoid arthritis. Pediatric Rheumatology Collaborative Study Group. N Engl J Med. 2000;342(11):763–9.

7. Lovell DJ, Giannini EH, Reiff A, Jones OY, Schneider R, Olson JC, et al. Long-term efficacy and safety of etanercept in children with polyarticular-course juvenile rheumatoid arthritis: interim results from an ongoing multicenter, open-label, extended-treatment trial. Arthritis Rheum. 2003;48(1):218–26.

8. Lovell DJ, Reiff A, Jones OY, Schneider R, Nocton J, Stein LD, et al. Long-term safety and efficacy of etanercept in children with polyarticular-course juvenile rheumatoid arthritis. Arthritis Rheum. 2006;54(6):1987–94.

9. Lovell DJ, Reiff A, Ilowite NT, Wallace CA, Chon Y, Lin SL, et al. Safety and efficacy of up to eight years of continuous etanercept therapy in patients with juvenile rheumatoid arthritis. Arthritis Rheum. 2008;58(5):1496–504.

10. Lovell DJ, Ruperto N, Goodman S, Reiff A, Jung L, Jarosova K, et al. Adalimumab with or without methotrexate in juvenile rheumatoid arthritis. N Engl J Med. 2008;359(8):810–20.

11. Lovell DJ, Brunner HI, Reiff AO, Jung L, Jarosova K, Němcová D, et al. Long-term outcomes in patients with polyarticular juvenile idiopathic arthritis receiving adalimumab with or without methotrexate. RMD Open. 2020;6(2):e001208.

12. Ruperto N, Lovell DJ, Cuttica R, Wilkinson N, Woo P, Espada G, et al. A randomized, placebo-controlled trial of infliximab plus methotrexate for the treatment of polyarticular-course juvenile rheumatoid arthritis. Arthritis Rheum. 2007;56(9):3096–106.

13. Ruperto N, Lovell DJ, Cuttica R, Woo P, Meiorin S, Wouters C, et al. Long-term efficacy and safety of infliximab plus methotrexate for the treatment of polyarticular-course juvenile rheumatoid arthritis: findings from an open-label treatment extension. Ann Rheum Dis. 2010;69(4):718–22.

14. Wallace CA, Giannini EH, Spalding SJ, Hashkes PJ, O'Neil KM, Zeft AS, et al. Trial of early aggressive therapy in polyarticular juvenile idiopathic arthritis. Arthritis Rheum. 2012;64(6):2012–21.

15. Constantin T, Foeldvari I, Vojinovic J, Horneff G, Burgos-Vargas R, Nikishina I, et al. Two-year efficacy and safety of etanercept in pediatric patients with extended oligoarthritis, enthesitis-related arthritis, or psoriatic arthritis. J Rheumatol. 2016;43(4):816–24.

16. Foeldvari I, Constantin T, Vojinović J, Horneff G, Chasnyk V, Dehoorne J, et al. Etanercept treatment for extended oligoarticular juvenile idiopathic arthritis, enthesitis-related arthritis, or psoriatic arthritis: 6-year efficacy and safety data from an open-label trial. Arthritis Res Ther. 2019;21(1):125.

17. Horneff G, Burgos-Vargas R, Constantin T, Foeldvari I, Vojinovic J, Chasnyk VG, et al. Efficacy and safety of open-label etanercept on extended oligoarticular juvenile idiopathic arthritis, enthesitis-related arthritis and psoriatic arthritis: part 1 (week 12) of the CLIPPER study. Ann Rheum Dis. 2014;73(6):1114–22.

18. Kingsbury DJ, Bader-Meunier B, Patel G, Arora V, Kalabic J, Kupper H. Safety, effectiveness, and pharmacokinetics of adalimumab in children with polyarticular juvenile idiopathic arthritis aged 2 to 4 years. Clin Rheumatol. 2014;33(10):1433–41.

19. Ruperto N, Brunner HI, Pacheco-Tena C, Louw I, Vega-Cornejo G, Spindler AJ, et al. Open-label phase 3 study of intravenous golimumab in patients with polyarticular juvenile idiopathic arthritis. Rheumatology (Oxford). 2021;60(10):4495–507.

20. Aeschlimann FA, Hofer KD, Cannizzaro Schneider E, Schroeder S, Lauener R, Saurenmann RK. Infliximab in pediatric rheumatology patients: a retrospective analysis of infusion reactions and severe adverse events during 2246 infusions over 12 years. J Rheumatol. 2014;41(7):1409–15.

21. Armaroli G, Klein A, Ganser G, Ruehlmann MJ, Dressler F, Hospach A, et al. Long-term safety and effectiveness of etanercept in JIA: an 18-year experience from the BiKeR registry. Arthritis Res Ther. 2020;22(1):258.

22. Alexeeva EI, Namazova-Baranova LS, Bzarova TM, Valieva SI, Denisova RV, Sleptsova TV, et al. Predictors of the response to etanercept in patients with juvenile idiopathic arthritis without systemic manifestations within 12 months: results of an open-label, prospective study conducted at the National Scientific and Practical Center of Children's Health, Russia. Pediatr Rheumatol Online J. 2017;15(1):51.

23. Alexeeva E, Dvoryakovskaya T, Denisova R, Sleptsova T, Isaeva K, Chomahidze A, et al. Dynamics of concomitant therapy in children with juvenile idiopathic arthritis treated with etanercept and methotrexate. Pediatr Neonatol. 2019;60(5):549–55.

24. Bader-Meunier B, Krzysiek R, Lemelle I, Pajot C, Carbasse A, Poignant S, et al. Etanercept concentration and immunogenicity do not influence the response to etanercept in patients with juvenile idiopathic arthritis. Semin Arthritis Rheum. 2019;48(6):1014–8.

25. Becker I, Horneff G. Risk of serious infection in juvenile idiopathic arthritis patients associated with tumor necrosis factor inhibitors and disease activity in the German Biologics in Pediatric Rheumatology registry. Arthritis Care Res (Hoboken). 2017;69(4):552–60.

26. Beukelman T, Xie F, Baddley JW, Chen L, Mannion ML, Saag KG, et al. The risk of hospitalized infection following initiation of biologic agents versus methotrexate in the treatment of juvenile idiopathic arthritis. Arthritis Res Ther. 2016;18(1):210.

27. Brunelli JB, Silva CA, Pasoto SG, Saa CGS, Kozu KT, Goldenstein-Schainberg C, et al. Anti-adalimumab antibodies kinetics: an early guide for juvenile idiopathic arthritis (JIA) switching. Clin Rheumatol. 2020;39(2):515–21.

28. Brunner HI, Nanda K, Toth M, Foeldvari I, Bohnsack J, Milojevic D, et al. Safety and effectiveness of adalimumab in patients with polyarticular course of juvenile idiopathic arthritis: STRIVE registry seven-year interim results. Arthritis Care Res (Hoboken). 2020;72(10):1420–30.

29. Cabrera N, Lega JC, Kassai B, Wouters C, Kondi A, Cannizzaro E, et al. Safety of biological agents in paediatric rheumatic diseases: a real-life multicenter retrospective study using the JIRcohorte database. Joint Bone Spine. 2019;86(3):343–50.

30. Choi JY, Chung JE, Park JH, Cho YS, Jung YW, Choi SA. Surveillance of adverse drug events associated with etanercept prescribed for juvenile idiopathic arthritis in a single center up to 9-years: a retrospective observational study. PLoS One. 2018;13(11):e0204573.

31. Davies R, Southwood TR, Kearsley-Fleet L, Lunt M, Hyrich KL. Medically significant infections are increased in patients with juvenile idiopathic arthritis treated with etanercept: results from the British Society for Paediatric and Adolescent Rheumatology Etanercept Cohort Study. Arthritis Rheumatol. 2015;67(9):2487–94.

32. Dumaine C, Bekkar S, Belot A, Cabrera N, Malik S, von Scheven A, et al. Infectious adverse events in children with juvenile idiopathic arthritis treated with biological agents in a real-life setting: data from the JIRcohorte. Joint Bone Spine. 2020;87(1):49–55.

33. Favalli EG, Pontikaki I, Becciolini A, Biggioggero M, Ughi N, Romano M, et al. Real-life 10-year retention rate of first-line anti-TNF drugs for inflammatory arthritides in adult- and juvenile-onset populations: similarities and differences. Clin Rheumatol. 2017;36(8):1747–55.

34. Feger DM, Longson N, Dodanwala H, Ostrov BE, Olsen NJ, June RR. Comparison of adults with polyarticular juvenile idiopathic arthritis to adults with rheumatoid arthritis: a cross-sectional analysis of clinical features and medication use. J Clin Rheumatol. 2019;25(4):163–70.

35. Finetti M, Giancane G, Bagnasco F, Dolezalova P, Tsitsami E, Trachana M, et al. Assessing the clinical relevance and risk minimization of antibodies to biologics in juvenile idiopathic arthritis (JIA) (ABIRISK) - preliminary results. Pediatr Rheumatol Online J. 2018;16:P191.

36. Geikowski T, Becker I, Horneff G. Predictors of response to etanercept in polyarticular-course juvenile idiopathic arthritis. Rheumatology (Oxford). 2014;53(7):1245–9.

37. Gerloni V, Pontikaki I, Gattinara M, Fantini F. Focus on adverse events of tumour necrosis factor alpha blockade in juvenile idiopathic arthritis in an open monocentric long-term prospective study of 163 patients. Ann Rheum Dis. 2008;67(8):1145–52.

38. Giannini EH, Ilowite NT, Lovell DJ, Wallace CA, Rabinovich CE, Reiff A, et al. Long-term safety and effectiveness of etanercept in children with selected categories of juvenile idiopathic arthritis. Arthritis Rheum. 2009;60(9):2794–804.

39. Glazyrina G, Sudareva O. 10-years experience of etanercept use in treatment of juvenile idiopathic atrtritis in Chelyabinsk regional pediatric hospital. Ann Rheum Dis. 2019;78:1957.

40. Goettel AM, DeClercq J, Choi L, Graham TB, Mitchell AA. Efficacy and safety of abatacept, adalimumab, and etanercept in pediatric patients with juvenile idiopathic arthritis. J Pediatr Pharmacol Ther. 2021;26(2):157–62.

41. Haapasaari J, Kautiainen H, Hannula S, Pohjankoski H, Hakala M. Good results from combining etanercept to prevailing DMARD therapy in refractory juvenile idiopathic arthritis. Clin Exp Rheumatol. 2002;20(6):867–70.

42. Halbig M, Horneff G. Improvement of functional ability in children with juvenile idiopathic arthritis by treatment with etanercept. Rheumatol Int. 2009;30(2):229–38.

43. Horneff G, Schmeling H, Biedermann T, Foeldvari I, Ganser G, Girschick HJ, et al. The German etanercept registry for treatment of juvenile idiopathic arthritis. Ann Rheum Dis. 2004;63(12):1638–44.

44. Horneff G, De Bock F, Foeldvari I, Girschick HJ, Michels H, Moebius D, et al. Safety and efficacy of combination of etanercept and methotrexate compared to treatment with etanercept only in patients with juvenile idiopathic arthritis (JIA): preliminary data from the German JIA Registry. Ann Rheum Dis. 2009;68(4):519–25.

45. Horneff G, Klein A, Klotsche J, Minden K, Huppertz HI, Weller-Heinemann F, et al. Comparison of treatment response, remission rate and drug adherence in polyarticular juvenile idiopathic arthritis patients treated with etanercept, adalimumab or tocilizumab. Arthritis Res Ther. 2016;18(1):272.

46. Horneff G, Schulz AC, Klotsche J, Hospach A, Minden K, Foeldvari I, et al. Experience with etanercept, tocilizumab and interleukin-1 inhibitors in systemic onset juvenile idiopathic arthritis patients from the BIKER registry. Arthritis Res Ther. 2017;19(1):256.

47. Horneff G, Minden K, Hospach T, Foeldvari I, Haas P, Thon A, et al. Efficacy and safety of adalimumab in juvenile idiopathic arthritis – 10 year experience using data of the biker registry. Ann Rheum Dis. 2019;78(Suppl 2):1340.

48. Horneff G, Zimmer A, Minden K, Hospach T, Weller-Heinemann F, Hansmann S, et al. Long-term observational safety surveillance of golimumab treatment for polyarticular juvenile idiopathic arthirtis—an interim analysis. Ann Rheum Dis. 2020;79:1207.

49. Kearsley-Fleet L, Davies R, Lunt M, Southwood TR, Hyrich KL. Factors associated with improvement in disease activity following initiation of etanercept in children and young people with juvenile idiopathic arthritis: results from the British Society for Paediatric and Adolescent Rheumatology Etanercept Cohort Study. Rheumatology (Oxford). 2016;55(5):840–7.

50. Kimura Y, Pinho P, Walco G, Higgins G, Hummell D, Szer I, et al. Etanercept treatment in patients with refractory systemic onset juvenile rheumatoid arthritis. J Rheumatol. 2005;32(5):935–42.

51. Klein A, Becker I, Minden K, Foeldvari I, Haas JP, Horneff G. Adalimumab versus adalimumab and methotrexate for the treatment of juvenile idiopathic arthritis: long-term data from the German BIKER registry. Scand J Rheumatol. 2019;48(2):95–104.

52. Klein A, Ganser G, Rühlmann M, Dressler F, Hospach T, Minden K, et al. Efficacy and safety of etanercept in juvenile idiopathic arthritis – 18 year experience using data of the BIKER registry. Ann Rheum Dis. 2019;78(Suppl 2):549.

53. Klein A, Becker I, Minden K, Hospach A, Schwarz T, Foeldvari I, et al. Biologic therapies in polyarticular juvenile idiopathic arthritis. comparison of long-term safety data from the German BIKER registry. ACR Open Rheumatol. 2020;2(1):37–47.

54. Klotsche J, Minden K, Thon A, Ganser G, Urban A, Horneff G. Improvement in health-related quality of life for children with juvenile idiopathic arthritis after start of treatment with etanercept. Arthritis Care Res (Hoboken). 2014;66(2):253–62.

55. Klotsche J, Niewerth M, Haas JP, Huppertz HI, Zink A, Horneff G, et al. Long-term safety of etanercept and adalimumab compared to methotrexate in patients with juvenile idiopathic arthritis (JIA). Ann Rheum Dis. 2016;75(5):855–61.

56. Klotsche J, Klein A, Niewerth M, Ganser G, Aries P, Walther M, et al. Safety profile of etanercept in long-term use in patients with juvenile idiopathic arthritis (JIA). Ann Rheum Dis. 2019;78:102.

57. Klotsche J, Horneff G, Haas P, Foeldvari I, Niewerth M, Minden K. Early implementation of treatment with etanercept increases the likelihood to achieve remission. Ann Rheum Dis. 2020;79:825.

58. McErlane F, Foster HE, Davies R, Lunt M, Watson KD, Symmons DP, et al. Biologic treatment response among adults with juvenile idiopathic arthritis: results from the British Society for Rheumatology Biologics Register. Rheumatology (Oxford). 2013;52(10):1905–13.

59. Minden K, Niewerth M, Zink A, Seipelt E, Foeldvari I, Girschick H, et al. Long-term outcome of patients with JIA treated with etanercept, results of the biologic register JuMBO. Rheumatology (Oxford). 2012;51(8):1407–15.

60. Nielsen S, Ruperto N, Gerloni V, Simonini G, Cortis E, Lepore L, et al. Preliminary evidence that etanercept may reduce radiographic progression in juvenile idiopathic arthritis. Clin Exp Rheumatol. 2008;26(4):688–92.

61. Otten MH, Prince FH, Anink J, Ten Cate R, Hoppenreijs EP, Armbrust W, et al. Effectiveness and safety of a second and third biological agent after failing etanercept in juvenile idiopathic arthritis: results from the Dutch National ABC Register. Ann Rheum Dis. 2013;72(5):721–7.

62. Otten MH, Prince FH, Armbrust W, ten Cate R, Hoppenreijs EP, Twilt M, et al. Factors associated with treatment response to etanercept in juvenile idiopathic arthritis. JAMA. 2011;306(21):2340–7.

63. Pastore S, Naviglio S, Canuto A, Lepore L, Martelossi S, Ventura A, et al. Serious adverse events associated with anti-tumor necrosis factor alpha agents in pediatric-onset inflammatory bowel disease and juvenile idiopathic arthritis in a real-life setting. Paediatr Drugs. 2018;20(2):165–71.

64. Prince FH, Geerdink LM, Borsboom GJ, Twilt M, van Rossum MA, Hoppenreijs EP, et al. Major improvements in health-related quality of life during the use of etanercept in patients with previously refractory juvenile idiopathic arthritis. Ann Rheum Dis. 2010;69(1):138–42.

65. Prince FH, Twilt M, ten Cate R, van Rossum MA, Armbrust W, Hoppenreijs EP, et al. Long-term follow-up on effectiveness and safety of etanercept in juvenile idiopathic arthritis: the Dutch national register. Ann Rheum Dis. 2009;68(5):635–41.

66. Quartier P, Taupin P, Bourdeaut F, Lemelle I, Pillet P, Bost M, et al. Efficacy of etanercept for the treatment of juvenile idiopathic arthritis according to the onset type. Arthritis Rheum. 2003;48(4):1093–101.

67. Romano M, Pontikaki I, Gattinara M, Ardoino I, Donati C, Boracchi P, et al. Drug survival and reasons for discontinuation of the first course of biological therapy in 301 juvenile idiopathic arthritis patients. Reumatismo. 2014;65(6):278–85.

68. Ruperto N, Spindler A, Pacheco Tena CF, Louw I, Cornejo GV, Kingsbury D, et al. Efficacy and safety of intravenous golimumab in patients with juvenile idiopathic arthritis: results from a phase 3 open-label study. Ann Rheum Dis. 2019;78:966.

69. Russo RA, Katsicas MM. Clinical remission in patients with systemic juvenile idiopathic arthritis treated with anti-tumor necrosis factor agents. J Rheumatol. 2009;36(5):1078–82.

70. Schmeling H, Minden K, Foeldvari I, Ganser G, Hospach T, Horneff G. Efficacy and safety of adalimumab as the first and second biologic agent in juvenile idiopathic arthritis: the German Biologics JIA Registry. Arthritis Rheumatol. 2014;66(9):2580–9.

71. Sevcic K, Orban I, Brodszky V, Bazso A, Balogh Z, Poor G, et al. Experiences with tumour necrosis factor-{alpha} inhibitors in patients with juvenile idiopathic arthritis: Hungarian data from the National Institute of Rheumatology and Physiotherapy Registry. Rheumatology (Oxford). 2011;50(7):1337–40.

72. Solari N, Palmisani E, Consolaro A, Pistorio A, Viola S, Buoncompagni A, et al. Factors associated with achievement of inactive disease in children with juvenile idiopathic arthritis treated with etanercept. J Rheumatol. 2013;40(2):192–200.

73. Southwood TR, Foster HE, Davidson JE, Hyrich KL, Cotter CB, Wedderburn LR, et al. Duration of etanercept treatment and reasons for discontinuation in a cohort of juvenile idiopathic arthritis patients. Rheumatology (Oxford). 2011;50(1):189–95.

74. Su Y, Yang YH, Chiang BL. Treatment response to etanercept in methotrexate refractory juvenile idiopathic arthritis: an analysis of predictors and long-term outcomes. Clin Rheumatol. 2017;36(9):1997–2004.

75. Takei S, Iwata N, Kobayashi I, Igarashi T, Yoshinaga Y, Matsubara N, et al. Safety and effectiveness of adalimumab in Japanese patients with juvenile idiopathic arthritis: results from a real-world postmarketing study. Mod Rheumatol. 2021;31(2):421–30.

76. Tambralli A, Beukelman T, Weiser P, Atkinson TP, Cron RQ, Stoll ML. High doses of infliximab in the management of juvenile idiopathic arthritis. J Rheumatol. 2013;40(10):1749–55.

77. Tarkiainen M, Tynjälä P, Vähäsalo P, Lahdenne P. Occurrence of adverse events in patients with JIA receiving biologic agents: long-term follow-up in a real-life setting. Rheumatology (Oxford). 2015;54(7):1170–6.

78. Trachana M, Pratsidou-Gertsi P, Badouraki M, Haidich AB, Pardalos G. Achievement of clinical remission in patients with juvenile idiopathic arthritis under a 2-10-year etanercept exposure. Clin Rheumatol. 2013;32(8):1191–7.

79. Tynjälä P, Vähäsalo P, Honkanen V, Lahdenne P. Drug survival of the first and second course of anti-tumour necrosis factor agents in juvenile idiopathic arthritis. Ann Rheum Dis. 2009;68(4):552–7.

80. Verazza S, Davì S, Consolaro A, Bovis F, Insalaco A, Magni-Manzoni S, et al. Disease status, reasons for discontinuation and adverse events in 1038 Italian children with juvenile idiopathic arthritis treated with etanercept. Pediatr Rheumatol Online J. 2016;14(1):68.

81. Verstegen R, Shrader P, Balevic S, Beukelman T, Correll C, Dennos A, et al. Variations in adalimumab and etanercept dosing in juvenile idiopathic arthritis and their effect on treatment outcome: a Childhood Arthritis and Rheumatology Research Alliance (CARRA) Registry study [abstract]. Arthritis Rheumatol. 2020;72.

82. Windschall D, Müller T, Becker I, Horneff G. Safety and efficacy of etanercept in children with the JIA categories extended oligoarthritis, enthesitis-related arthritis and psoriasis arthritis. Clin Rheumatol. 2015;34(1):61–9.

83. Windschall D, Horneff G. Safety and efficacy of etanercept and adalimumab in children aged 2 to 4 years with juvenile idiopathic arthritis. Clin Rheumatol. 2016;35(12):2925–31.

84. Zuber Z, Rutkowska-Sak L, Postępski J, Dobrzyniecka B, Opoka-Winiarska V, Kobusińska K, et al. Etanercept treatment in juvenile idiopathic arthritis: the Polish registry. Med Sci Monit. 2011;17(12):SR35–42.

85. Kearsley-Fleet L, Davies R, Baildam E, Beresford MW, Foster HE, Southwood TR, et al. Factors associated with choice of biologic among children with Juvenile Idiopathic Arthritis: results from two UK paediatric biologic registers. Rheumatology (Oxford). 2016;55(9):1556–65.

86. Klein A, Klotsche J, Hügle B, Minden K, Hospach A, Weller-Heinemann F, et al. Long-term surveillance of biologic therapies in systemic-onset juvenile idiopathic arthritis: data from the German BIKER registry. Rheumatology (Oxford). 2020;59(9):2287–98.
